# Computational insights on the molecular interplay between KRas (G12D mutation) and SOS1 modulated by the inhibitor BI-3406

**DOI:** 10.1371/journal.pcbi.1014213

**Published:** 2026-04-29

**Authors:** Juan Zeng, Yixuan Lan, Fei Xia

**Affiliations:** 1 School of Biomedical Engineering, Guangdong Medical University, Dongguan, China; 2 Shanghai Engineering Research Center of Molecular Therapeutics and New Drug Development, School of Chemistry and Molecular Engineering, East China Normal University, Shanghai, China; 3 NYU-ECNU Center for Computational Chemistry at NYU Shanghai, Shanghai, China; Aarhus University, DENMARK

## Abstract

Ras proteins are prominent oncogenes, with KRas mutations found in approximately 80% of cancer cells harboring Ras mutations. The mechanism by which Ras mutations cause cancer remains unclear. Human Son of Sevenless (SOS) promotes the GDP-to-GTP exchange in the inactive GDP-bound Ras (RasGDP) by interacting with RasGDP conformation, thereby leading to the development of human cancer. Elucidating the Ras-SOS interaction mechanism can guide the drug design for Ras and SOS proteins. Based on our previously sampled special structure KRasGDP·Mg^2+^_S1.2_, this study constructs a functional ternary complex (KRasGDP·Mg^2+^)·SOS1·(KRasGTP·Mg^2+^). Furthermore, the KRas-SOS1 interactions regulated by the KRas G12D mutation and the SOS1 inhibitor BI-3406 that reportedly exhibits synergistic effects with G12D-mutant Ras inhibitors, are investigated through molecular dynamics (MD) simulations. The findings reveal that the G12D mutation and BI-3406 both affect the KRas-SOS1 interaction via the Switch-II (SW2) region of KRas. The negatively charged Asp12 has a repulsive effect on KRas, particularly on SW2, altering the interfacial electrostatic landscapes and diminishing the binding affinities by approximately 25 kcal/mol for both KRasGDP·Mg^2+^ and KRasGTP·Mg^2+^. BI-3406 forms a hydrogen-bond bridge between SW2 and SOS1 in wild type (WT) KRas, interrupting the interactions among the N-terminal residues of SW2 and SOS1. Moreover, BI-3406 was found here to attenuate the binding affinity of both WT and G12D-mutant KRasGDP·Mg^2+^ to SOS1. Interestingly, BI-3406 hardly affects the binding affinity of WT KRasGTP·Mg^2+^, while enhances the binding affinity of G12D-mutant KRasGTP·Mg^2+^. The change of binding affinity makes the catalytic pocket of SOS1 prefer to KRasGTP·Mg^2+^ and inhibits the growth of G12D-mutant KRas-driven tumors. These mechanistic insights provide valuable information for designing SOS1-co-targeting inhibitors to potentiate antitumor efficacy against G12D-mutated KRas.

## Author summary

Protein-protein interactions serve as the crucial cornerstone for constructing cellular signaling pathways. Drug design targeted at protein interaction interfaces has emerged as the main strategy for treating human diseases. Dysfunction of Ras signaling pathway is associated with various human tumors, making Ras proteins the key drug target. inhibitors such as Sotorasib targeting G12C mutated KRas can treat the non-small-cell lung cancer. However, the emergence of drug resistance poses a significant challenge to Ras inhibitors, making the upstream regulatory factor SOS1 a potential target to overcome the bottleneck. BI-3406, as the first orally effective selective SOS1 inhibitor, demonstrates significant antitumor activity in tumor models with KRas G12C, G12D, G12V, and G13D mutations, and exhibits synergistic antitumor effects when combined with KRas inhibitors. This work provides the computational insights on the molecular interplay between KRas (G12D Mutation) and SOS1 modulated by the inhibitor BI-3406, which is valuable for Ras-related drug design.

## 1. Introduction

**Fig 1:**
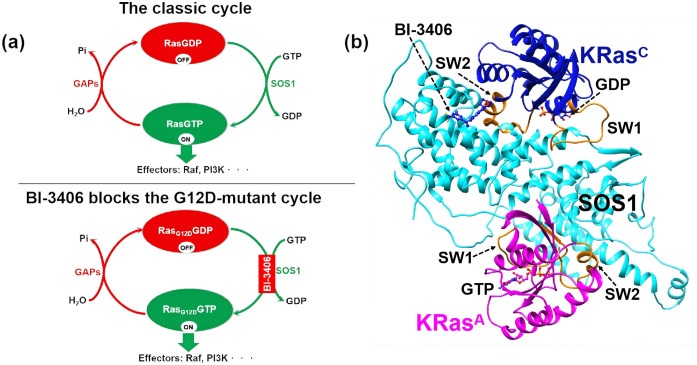
The GDP-to-GTP exchange cycle and structure of KRas regulated by G12D mutation and SOS1 inhibitor BI-3406. (a) SOS1 inhibitor BI-3406 interrupts the GDP-to-GTP exchange cycle in G12D-mutant Ras protein. (b) The (KRas^C^GDP·Mg^2+^)·SOS1·(KRas^A^GTP·Mg^2+^) functional ternary complex with SOS1 inhibitor BI-3406. GDP, GTP and BI-3406 are shown in stick-ball model. The SW1 and SW2 regions of KRas are highlighted with orange. The superscripted KRas^C^GDP·Mg^2+^ represents KRas within the catalytic site of SOS1, and superscripted KRas^A^GDP·Mg^2+^ represents KRas within the allosteric site of SOS1.

In Ras-Raf-MEK-ERK signaling pathway, extracellular signaling molecules trigger activation of the Son of Sevenless (SOSs) family (SOS1 and SOS2) [1–3], one of the guanosine nucleotide exchange factors (GEFs). The activated SOSs engage the GDP-bound Ras isoforms (HRas, KRas, and NRas), catalyzing the replacement of bound GDP in Ras proteins with GTP in solvent (Fig 1a) [4–7]. The GDP-to-GTP exchange converts Ras proteins from an inactive “OFF” state (GDP-bound state represented by RasGDP) to an active “ON” state (GTP-bound state represented by RasGTP). Activated Ras proteins propel downstream effectors like Raf kinase [8–11], orchestrating cellular responses to environmental change. The Ras signaling pathway participates in cell proliferation and survival, differentiation, apoptosis, cytoskeletal movement, protein transport and secretion [12–15]. Dysregulation of Ras signaling pathway is inextricably linked to oncogenesis [16,17]. The analysis of data from TCGA and various tumor databases reveal there are about 20% of human cancers with one or multiple Ras mutations [18,19]. This underscores the importance of proteins within the Ras signaling pathway as significant drug targets, with Ras proteins and SOSs emerging as particularly promising cancer drug targets.

KRas mutants are detected in ~ 80% Ras-mutant cancer cells [19]. Notably, approximately 83% of KRas mutations cluster at residue Gly12 within the P-loop region (residues 10–17) [20,21]. The mechanism by which Ras mutations cause cancer is highly complex. On one hand, the mutations affect the Ras conformation; on the other hand, they also influence the interaction between Ras and the effector such as Raf and SOS1. The lack of understanding the mechanism by which Ras mutations cause cancer seriously affects the progress of drug design. To date, there are only two FDA approved Ras-targeted drugs: Sotorasib (AMG510) [22] and Adagrasib (MRTX849) [23], both covalent inhibitors specific to the KRas G12C mutant. Sotorasib and Adagrasib keeps the switch-I (SW1, residues 25–40) and switch-II (SW2, residues 58–74) regions in an “open” conformation [24] with a binding pocket (S1 Fig). However, the G12D mutant, which accounts for ~81% of KRas mutations, currently has no approved drug. The superimposed structures suggest a similar binding pocket in the KRas G12D mutant (S1 Fig) [25–29], inspiring the design of G12D KRas inhibitors [26–28,30], particularly most of that form salt bridge with D12 [26,28]. Numerous studies have reported that SOS1 inhibitors can enhance the inhibitory efficacy of Ras inhibitors. For example, SOS1 inhibitor MRTX0902 significantly enhances the tumor-suppressing capability of KRas inhibitor Adagrasib [23]. Both Ras and SOS inhibitors affect the GDP-to-GTP exchange activity [17,29,31–39]. Studies reveal there is dominant role of SOS1 over SOS2 in Ras activation [40]. Current researches concentrate on developing SOS1-specific inhibitors, with SOS2 inhibitors only emerging in 2024 [36]. Much SOS1 inhibitors–including BAY-293 [33,38], MRTX0902 [37], and 13c [32]—show efficacy against the KRas G12C mutant. They can synergize with G12C-specific inhibitors to enhance antitumor responses. In 2024, Duo et al. leveraged machine learning methods to discover SOS1 inhibitors targeting G12C mutated KRas/SOS1 interface [41]. Inhibitors targeting the Ras/SOS1 interface [42–47] disrupt the Ras-SOS1 interactions, trapping Ras in the inactive RasGDP state and thereby suppressing the tumors. BI-3406 stands out as the sole SOS1 inhibitor effective across prevalent KRas mutants (G12D, G12V, G12C, G13D) [34]. Its derivative, BI-1701963, now progresses through phase 1 clinical trials.

The conformation of Ras and SOS1 both influence Ras-SOS interactions [48]. The previous studies have uncovered the transition mechanisms [49–52], the conformational functions of the substates of Ras [20,24,53–56] and proposed the specific structures of Ras in complex with SOS1 [5,57–61]. SOS1, consisting of 1333 amino acids, organizes into six functional domains [59,62–65]. The REM and CDC25 domains of SOS1 both contain Ras-binding sites, enabling SOS1 to form the ternary complex (Fig 1b). The CDC25 domain has the catalytic site, preferentially engaging GDP-bound KRas (KRasGDP) to accelerate the GDP-to-GTP exchange. Most works focus on the KRas·SOS1 binary complex to studying the interaction between KRas and CDC25 domain, hoping to reveal the mechanism by which SOS1 promotes the GDP-GTP exchange [38,58,66,67]. The REM domain serves as the allosteric site, exhibiting stronger binding affinity for GTP-bound KRas (KRasGTP) [62,68]. The REM domain further binds with KRasGTP to form a (KRasGDP·Mg^2+^)·SOS1·(KRasGTP·Mg^2+^) ternary complex, which can synergistically enhance the efficiency of GDP-to-GTP exchange [5,69]. Margarit et al. have reported the ternary crystal structure (PDB ID 1NVW) formed with SOS1 and HRas [5]. There are several experimental HRas·SOS·HRas ternary complex [44–46,62,70]. However, all experimentally reported HRas·SOS·HRas ternary complexes are not the HRasGDP·SOS·HRasGTP functional state. Moreover, only one experimental ternary complex of G13D KRas mutant with SOS1 has been reported (PDB ID 7KFZ) [58]. This complex features KRas in its nucleotide-free form at the CDC25 domain. Until now, there is no reported RasGDP·SOS·RasGTP ternary complex structure.

This work constructs a functional ternary complex (KRasGDP·Mg^2+^)·SOS1·(KRasGTP·Mg^2+^). It has been reported that Ras mutations and partner binding will lead to conformational changes and promote GDP extraction [67,71,72], which can also affect the Ras-SOS interactions. So, we exclusively examine the impact of the KRas G12D mutation and SOS1 inhibitor BI-3406 on the SOS1-KRas interactions by molecular dynamic (MD) simulation. Based on the experimental structure 7KFZ, the (KRas^C^GDP·Mg^2+^)·SOS1·(KRas^A^GTP·Mg^2+^) and (KRas^C^GTP·Mg^2+^)·SOS1·(KRas^A^GTP·Mg^2+^) ternary complex with KRas G12D mutation or SOS1 inhibitor BI-3406 are constructed. Here, the superscript C in KRas^C^GDP·Mg^2+^ and KRas^C^GTP·Mg^2+^ refers to KRas bound at the catalytic site of the CDC25 domain, and the superscript A in KRas^A^GTP·Mg^2+^ represents KRas bound at the allosteric site of the REM domain. The results prove that our constructed ternary complexes can be stable during 1 µs MD trajectory. The pair-wise interactions show that the SW1 and SW2 regions of KRas play the key role for SOS1 binding, especially residues Tyr32, Glu57 and Gln61. The predicted electrostatic potential (ESP) indicates that the G12D mutation obviously alters the interfacial electrostatic landscapes, especially SW2 region that has repulsive effect with Asp12. In the representative structures, BI-3406 bridges SOS1 with KRas through a hydrogen-bond network at the C-termini of SW2 region, which introduce the flexibility into the KRas-SOS1 interface. The results of binding affinity show that BI-3406 reduces the binding affinity of both WT and G12D-mutant KRasGDP·Mg^2+^ to SOS1. However, BI-3406 hardly reduces the binding affinity of WT KRasGTP·Mg^2+^ while increasing the binding affinity of G12D-mutant KRasGTP·Mg^2+^, thereby making the catalytic pocket of SOS1 prefer to interact with KRasGTP·Mg^2+^ and disruppting the classic GDP-to-GTP exchange (Fig 1a). This interruption increases the probability of the inactive conformation (KRasGDP·Mg^2+^) and ultimately inhibits the growth of G12D-mutant KRas-driven tumors. The atomic structure and binding affinity both hint the synergistic effects of G12D mutation and BI-3406 on the KRas-SOS1 interaction.

## 2. Results and discussions

### 2.1. The constructed ternary complexes are stable

**Fig 2:**
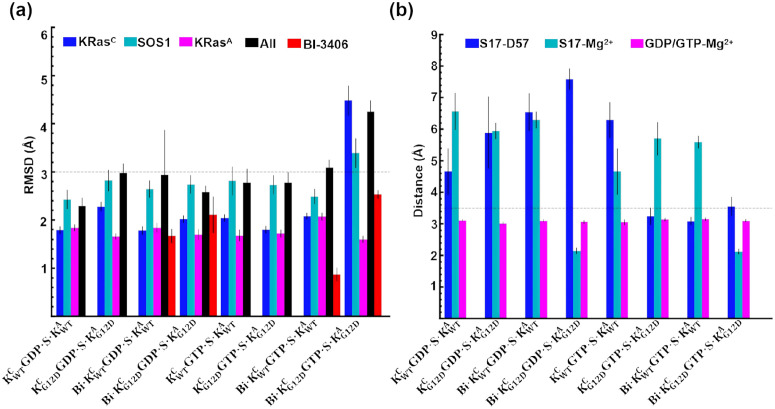
The RMSD values and coordinates within the binding pocket of KRas exhibite the stability. (a) The mean RMSD values with standard deviations indicate the stability of the complex, with the labels KRas^C^, SOS1, KRas^A^, All, and BI-3406 representing the RMSD of KRas at the catalytic site; SOS1 excluding the long loop (residues R744-L779); KRas at the allosteric site; the entire system (including GDP, GTP, and BI-3406) excluding the long loop regions of SOS1; and BI-3406, respectively. (b) The mean distances with standard deviations reveal the dynamics of Mg^2+^ coordination in KRas^C^, including the S17-D57 distance (Ser17 OG atom to the center of OD1, OD2 atoms in Asp57), the S17-Mg^2+^ distance (Ser17 OG atom to Mg^2+^ atom), and the GDP/GTP-Mg^2+^ distance (P_β_ atom of GDP or GTP to Mg^2+^).

The WT functional ternary complex (KRas^C^GDP·Mg^2+^)·SOS1·(KRas^A^GTP·Mg^2+^) was abbreviated as $\overset{\text{C}}{\underset{\text{WT}}{\text{K}}}\text{GDP}\cdot$S$\cdot \overset{\text{A}}{\underset{\text{WT}}{\text{K}}}$ (Fig 1b). After the nucleotide exchange, KRasGTP·Mg^2+^ occupies the catalytic site, yielding (KRas^C^GTP·Mg^2+^)·SOS1·(KRas^A^GTP·Mg^2+^) complex designated as $\overset{\text{C}}{\underset{\text{WT}}{\text{K}}}\text{GTP}\cdot$S$\cdot \overset{\text{A}}{\underset{\text{WT}}{\text{K}}}$. S2 Fig illustrates the time evolution of the heavy-atom RMSD for each ternary complex from three independent simulations. All RMSD values remained below 5 Å with small fluctuation, indicating the stability of our constructed ternary complexes. Fig 2a presents the mean RMSD values and standard deviation (STD) for each component within the ternary complex, calculated over the last 200-ns trajectory. KRas^A^ with RMSD values below 2 Å was stable in all complexes, while G12D mutants exhibit marginally reduced RMSD fluctuations, suggesting mutation-induced structural stabilization. SOS1 maintains stable conformations with RMSD values less than 3 Å except in the long loop region (residues A743-H780). Notably, Bi·$\overset{\text{C}}{\underset{\text{G12D}}{\text{K}}}\text{GTP}\cdot$S$\cdot \overset{\text{A}}{\underset{\text{G12D}}{\text{K}}}$ displays pronounced SOS1 flexibility, potentially linked to the substantial RMSD fluctuations of $\overset{\text{C}}{\underset{\text{G12D}}{\text{KRas}}}$ approaching 5 Å. In other complexes, the RMSD values of KRas^C^ fluctuate around 2 Å. The results highlight G12D mutation’s destabilizing effect on KRas^C^, particularly evident in Bi·$\overset{\text{C}}{\underset{\text{G12D}}{\text{K}}}\text{GTP}\cdot$S$\cdot \overset{\text{A}}{\underset{\text{G12D}}{\text{K}}}$. The RMSD values of BI-3406 fluctuated ~ 2 Å, but G12D mutation-induced perturbations emerge clearly in KRas^C^GTP·Mg^2+^-bound states, with RMSD increasing from 1 Å in Bi·$\overset{\text{C}}{\underset{\text{WT}}{\text{K}}}\text{GTP}\cdot$S$\cdot \overset{\text{A}}{\underset{\text{WT}}{\text{K}}}$ to 2.5 Å in Bi·$\overset{\text{C}}{\underset{\text{G12D}}{\text{K}}}\text{GTP}\cdot$S$\cdot \overset{\text{A}}{\underset{\text{G12D}}{\text{K}}}$. Crucially, each component within all ternary complexes maintain RMSD values below 5 Å with small fluctuation, confirming system stability regardless of G12D mutation status or BI-3406 presence.

### 2.2. The structural features of KRas at the catalytic site of SOS1 (KRas^C^) are dynamic

Our previous works identified at least two stable special conformations within KRasGDP·Mg^2+^ state 1, among which KRasGDP·Mg^2+^_S1.2_ interacts with SOS1 [54]. The cluster analysis was performed on the last 200 ns trajectory to extract representative structures of each ternary complex. For comparison with the specific KRasGDP·Mg^2+^_S1.2_ conformation, the representative structures of KRas^C^ were projected onto our previous two-dimensional free energy landscape (2D-FEL) (S3a Fig). Notably, all Rg values of KRas^C^ in ternary complex are slightly smaller than those of KRasGDP·Mg^2+^_S1.2_, indicating a more compact structure of KRas^C^ in the complex. The representative structures reveal that the larger Rg values stem from conformational changes in SW1, which adopts a more open conformation in the SOS1-bound state compared to KRasGDP·Mg^2+^_S1.2_ (S4 Fig). Except for Bi·$\overset{\text{C}}{\underset{\text{G12D}}{\text{K}}}\text{GTP}\cdot$S$\cdot \overset{\text{A}}{\underset{\text{G12D}}{\text{K}}}$, all RMSD values are also slightly smaller that those of KRasGDP·Mg^2+^_S1.2_. Bi·$\overset{\text{C}}{\underset{\text{G12D}}{\text{K}}}\text{GTP}\cdot$S$\cdot \overset{\text{A}}{\underset{\text{G12D}}{\text{K}}}$ exhibits increased RMSD (6.1 Å) and Rg (15.4 Å) values, suggesting a large conformational change in which SW1 completely open (S4 Fig). Excluded Bi·$\overset{\text{C}}{\underset{\text{G12D}}{\text{K}}}\text{GTP}\cdot$S$\cdot \overset{\text{A}}{\underset{\text{G12D}}{\text{K}}}$, pairwise RMSD values of KRas^C^ fluctuate around 2 Å (S3b Fig). Further analysis revealed that the substantial conformational shift in Bi·$\overset{\text{C}}{\underset{\text{G12D}}{\text{K}}}\text{GTP}\cdot$S$\cdot \overset{\text{A}}{\underset{\text{G12D}}{\text{K}}}$ primarily stems from SW1 fluctuations. Intriguingly, the enhanced SW1 dynamics in SOS1-free G12D KRas mutant were also observed by our previous work, attributed to the strong repulsive interactions between D12 and D33 within SW1 [73].

The Mg^2+^ ion establishes a six-coordinate configuration with GDP or GTP (S5 Fig). The prior research reveals that the α-helix (residues F929-N944) of SOS1 insertion into the active site of HRas disrupts the six-coordination [57]. In KRas^C^GDP·Mg^2+^ ternary complexes, the S17-D57 hydrogen bond was broken (Fig 2b). The S17-Mg^2+^ ion coordination only persists in Bi·$\overset{\text{C}}{\underset{\text{G12D}}{\text{K}}}\text{GDP}\cdot$S$\cdot \overset{\text{A}}{\underset{\text{G12D}}{\text{K}}}$, while being disrupted in three other complexes. However, the Mg^2+^ ion always coordinates with GDP, suggesting the coupled dissociation of GDP with Mg^2+^ ion. In KRas^C^GTP·Mg^2+^ ternary complexes, the S17-D57 hydrogen bond was preserved, except in $\overset{\text{C}}{\underset{\text{WT}}{\text{K}}}\text{GTP}\cdot$S$\cdot \overset{\text{A}}{\underset{\text{WT}}{\text{K}}}$. However, the S17-Mg^2+^ ion coordination was broken, except in Bi·$\overset{\text{C}}{\underset{\text{G12D}}{\text{K}}}\text{GTP}\cdot$S$\cdot \overset{\text{A}}{\underset{\text{G12D}}{\text{K}}}$. Notably, Mg^2+^ ion always coordinates with GTP. Within Bi·$\overset{\text{C}}{\underset{\text{G12D}}{\text{K}}}\text{GTP}\cdot$S$\cdot \overset{\text{A}}{\underset{\text{G12D}}{\text{K}}}$, this three-coordination remains intact. This interaction network among GTP, Mg^2+^ ion, S17, and D57 facilitates GTP binding at active site of KRas following GDP release.

**Fig 3:**
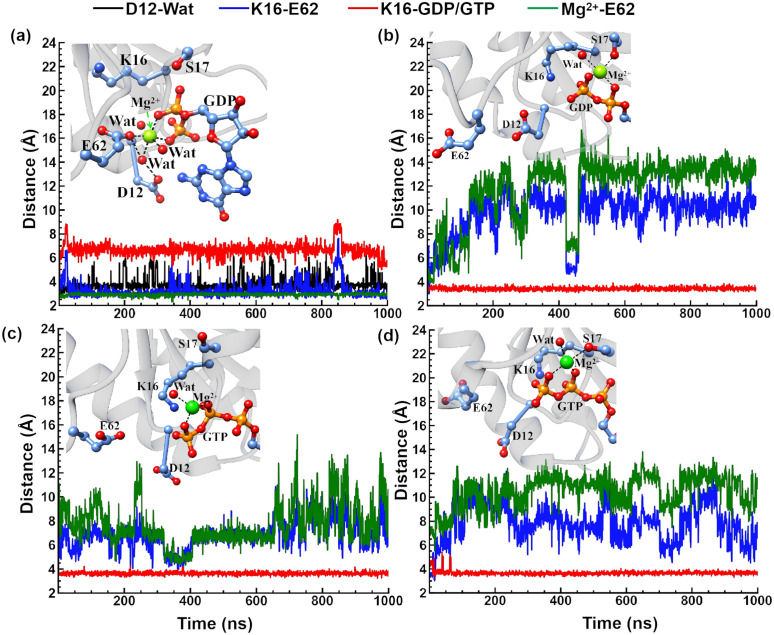
The distances of the Mg^2+^ ion cooridnates within G12D mutant KRas^C^ ternary complex are illustrated. The distances of the Mg^2+^ ion cooridnates within G12D mutant KRas^C^ ternary complex are illustrated. The distances are shown as functions of simulation time for $\begin{array}{c} \overset{\text{C}}{\underset{\text{G12D}}{\text{K}}}\text{GDP}\cdot \end{array}$S$\begin{array}{c} \cdot \overset{\text{A}}{\underset{\text{G12D}}{\text{K}}} \end{array}$ (a), Bi·$\begin{array}{c} \overset{\text{C}}{\underset{\text{G12D}}{\text{K}}}\text{GDP}\cdot \end{array}$S$\begin{array}{c} \cdot \overset{\text{A}}{\underset{\text{G12D}}{\text{K}}} \end{array}$ (b), $\begin{array}{c} \overset{\text{C}}{\underset{\text{G12D}}{\text{K}}}\text{GTP}\cdot \end{array}$S$\begin{array}{c} \cdot \overset{\text{A}}{\underset{\text{G12D}}{\text{K}}} \end{array}$ (c), and Bi·$\begin{array}{c} \overset{\text{C}}{\underset{\text{G12D}}{\text{K}}}\text{GTP}\cdot \end{array}$S$\begin{array}{c} \cdot \overset{\text{A}}{\underset{\text{G12D}}{\text{K}}} \end{array}$ (d).These include the D12-Wat distance (between the center of Asp12 OD1, OD2 atoms and the O atom of its coordinating water), the K16-E62 distance (between Lys16 NZ atom and the center of Glu62 OE1, OE2 atoms), the K16-GDP/GTP distance (between Lys16 NZ atoms and PB atom in GDP or GTP), and the Mg^2+^-E62 distance (between the Mg^2+^ ion and the center of Glu62 OE1, OE2 atoms).

The G12D mutation introduces one negative atomic charge, thereby reorganizing the Mg^2+^ ion coordination. In $\overset{\text{C}}{\underset{\text{G12D}}{\text{K}}}\text{GDP}\cdot$S$\cdot \overset{\text{A}}{\underset{\text{G12D}}{\text{K}}}$, the D12 side chain coordinates Mg^2+^ ion via a water bridge that simultaneously forms a hydrogen bond with the E62 side chain. Notably, the E62 side chain also coordinates Mg^2+^ ion while establishing a robust salt bridge with K16, causing the K16 side chain to reorient away from GDP (Fig 3). The Mg^2+^ ion further coordinates two water molecules and remains chelated by oxygen atoms of the diphosphate group in GDP. The six-coordination structure in $\overset{\text{C}}{\underset{\text{G12D}}{\text{K}}}\text{GDP}\cdot$S$\cdot \overset{\text{A}}{\underset{\text{G12D}}{\text{K}}}$ gathers negative charges from D12, E62, and GDP largely exceeding the neutralizing capacity of Mg^2+^ ion, introducing substantial fluctuations of GDP at the KRas^C^ active site (S6 Fig). It is consistent with the conclusion found by Hu et al. that the G12D mutation will accelerates the rate of GDP extraction [67]. In all three independent trajectories of $\overset{\text{C}}{\underset{\text{G12D}}{\text{K}}}\text{GDP}\cdot$S$\cdot \overset{\text{A}}{\underset{\text{G12D}}{\text{K}}}$, GDP exhibits obvious fluctuations. Structural analysis reveals that this reflects a notable positional shift of the purine ring relative to its orientation in the other three systems. Interesting, in two of three trajectories for Bi·$\overset{\text{C}}{\underset{\text{WT}}{\text{K}}}\text{GTP}\cdot$S$\cdot \overset{\text{A}}{\underset{\text{WT}}{\text{K}}}$, the phosphate groups of GTP exhibited significant relative movement compared to those in the other three systems. In the other three G12D mutated complexes, the K16-E62 salt bridge was disrupt, redirecting the K16 side chain persistently toward GDP or GTP. The distance between the NZ atom of K16 and PB atom of GDP/GTP is less than 4 Å (Fig 3). This strong electrostatic effect from K16 stabilizes GDP or GTP and reduces the RMSD values below 2 Å (S6 Fig). Similarly, the E62-Mg^2+^ ion coordination was also disrupt in other three systems. Intriguingly, Bi·$\overset{\text{C}}{\underset{\text{G12D}}{\text{K}}}\text{GDP}\cdot$S$\cdot \overset{\text{A}}{\underset{\text{G12D}}{\text{K}}}$ and Bi·$\overset{\text{C}}{\underset{\text{G12D}}{\text{K}}}\text{GTP}\cdot$S$\cdot \overset{\text{A}}{\underset{\text{G12D}}{\text{K}}}$ preserve Mg^2+^ ion coordination with S17—a feature absent in BI-3406 free state (Fig 2b).

### 2.3. The interactions between KRas^C^ and SOS1 are affected by G12D mutation and BI-3406

This section focuses on searching the interactions between KRas^C^ and SOS1. The pair-wise residue distances with the heavy atoms in side chain between KRas^C^ and SOS1 are calculated to find the interactions. The interactions are defined as residue pairs maintaining a spatial proximity of less than 6 Å.

#### 2.3.1. G12D mutation and BI-3406 affect the interactions between KRas^C^GDP·Mg^2+^ and SOS1.

**Fig 4:**
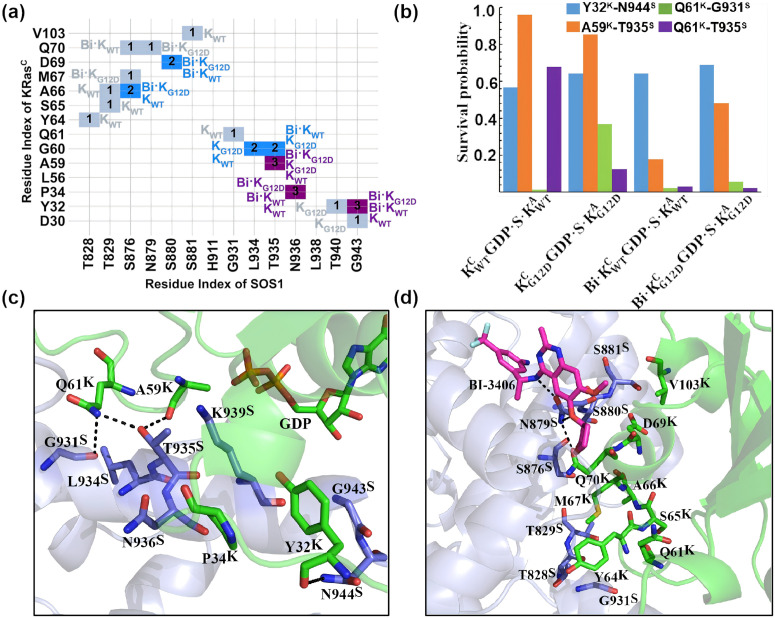
The overall interactions between KRas^C^GDP·Mg^2+^ and SOS1 are summarized. (a) The interaction matrix between KRas^C^ and SOS1 is shown for the four KRas^C^GDP·Mg^2+^ ternary complexes. K_WT_ represents the results of $\overset{\text{C}}{\underset{\text{WT}}{\text{K}}}\text{GDP}\cdot$S$\cdot \overset{\text{A}}{\underset{\text{WT}}{\text{K}}}$. K_G12D_ shows the results of $\overset{\text{C}}{\underset{\text{G12D}}{\text{K}}}\text{GDP}\cdot$S$\cdot \overset{\text{A}}{\underset{\text{G12D}}{\text{K}}}$. Bi·K_WT_ is the results of Bi·$\overset{\text{C}}{\underset{\text{WT}}{\text{K}}}\text{GDP}\cdot$S$\cdot \overset{\text{A}}{\underset{\text{WT}}{\text{K}}}$. Bi·K_G12D_ describes the results of Bi·$\overset{\text{C}}{\underset{\text{G12D}}{\text{K}}}\text{GDP}\cdot$S$\cdot \overset{\text{A}}{\underset{\text{G12D}}{\text{K}}}$. The number ‘1’, ‘2’, ‘3’ and ‘4’ indicate that an interaction was observed in one, two, three and four of the simulated complexes, respectively. (b) The survival probability of key hydrogen bonds are shown as a bar graph. Here, the superscript “K” in Y32^K^, A59^K^ and Q61^K^ denotes residues from KRas^C^, while the superscript “S” in G931^S^, T935^S^ and N944^S^ denotes residues from SOS1. (c, d) the atomic interactions between KRas^C^ and SOS1, viewed from two distinct perspectives. The key residues are shown in stick-ball model. The dashed lines shows the hydrogen bonds.

The pairwise distance analysis reveals that SW1 and SW2 are the primary contributors mediating interactions between KRas^C^ and SOS1 (Fig 4a). In SW1, residues Y32 and P34 of WT KRas^C^ (Y32^K^, P34^K^) engage in hydrophobic interactions with residues N936 and G943 of SOS1 (N936^S^, G943^S^)—a feature notably absent in $\overset{\text{C}}{\underset{\text{G12D}}{\text{K}}}\text{GDP}\cdot$S$\cdot \overset{\text{A}}{\underset{\text{G12D}}{\text{K}}}$. The structure shows that the backbone atom of Y32^K^ forms one hydrogen bond with N944^S^ (Fig 4b and 4c). The Y32^K^-N944^S^ hydrogen bond remains highly stable in all KRas^C^GDP·Mg^2+^ complexes, it remains stable for long than 500 ns during the entire simulation time. (Fig 4b).

In SW2, the pairwise distance analysis reveals that A59^K^ establishes stable interaction with T935^S^ within the α-helix of SOS1. Structural analysis uncovers one hydrogen bond network involving A59^K^, Q61^K^, G931^S^ and T935^S^. Specifically, the backbone atom O of A59^K^ forms one hydrogen bond with the side chain atom OG of T935^S^ (Fig 4c). The A59^K^-T935^S^ hydrogen bond is stable in the ternary complex without BI-3406. However, BI-3406 binding heavily affect the A59^K^-T935^S^ hydrogen bond. The survival probability is significantly reduced, with a notable reduction observed in Bi·$\begin{array}{c} \overset{\text{C}}{\underset{\text{WT}}{\text{K}}}\text{GDP}\cdot \end{array}$S$\cdot \overset{\text{A}}{\underset{\text{WT}}{\text{K}}}$(Fig 4b). Simultaneously, T935^S^ forms one stable hydrogen bond with the side chain of Q61^K^ in $\overset{\text{C}}{\underset{\text{WT}}{\text{K}}}\text{GDP}\cdot$S$\cdot \overset{\text{A}}{\underset{\text{WT}}{\text{K}}}$—a interaction exhibiting significant fluctuations in other three ternary complexes. Furthermore, the side chain of Q61^K^ forms one hydrogen bond with the backbone atom of G931^S^ for a very short duration. The mutual effect of the G12D mutation and BI-3406 binding profoundly destabilizes the Q61^K^-T935^S^ hydrogen bond.

In SW2, the N-terminal residues Y64^K^, S65^K^ and A66^K^ prefer to interact with T828^S^, T829^S^ and S876^S^ in $\overset{\text{C}}{\underset{\text{WT}}{\text{K}}}\text{GDP}\cdot$S$\cdot \overset{\text{A}}{\underset{\text{WT}}{\text{K}}}$ (Fig 4a and 4d). Both G12D mutation and BI-3406 interrupt the interactions. The N-terminal charged residues E62^K^ and E63^K^ has a repulsive effect with D12^K^. With or without BI-3406, the distance between CA atom of D12^K^/G12^K^ and the geometrical center of OE1, OE2 atoms in E62^K^ from the G12D mutated KRas^C^GDP·Mg^2+^ ternary complex is always significantly larger than that in WT (S7 Fig). The C-terminal residues M67^K^, D69^K^ and Q70^K^ tend to approach S876^S^, N879^S^ and S880^S^ at BI-3406 bound ternary complexes (Fig 4a and 4d). The atomic structure of Bi·$\overset{\text{C}}{\underset{\text{WT}}{\text{K}}}\text{GDP}\cdot$S$\cdot \overset{\text{A}}{\underset{\text{WT}}{\text{K}}}$ reveals that the C-terminal residues Q70^K^ and R73^K^ participate in forming the binding pocket of BI-3406. The detailed interactions between BI-3406 and the ternary complexes will be described in **section 2.3.4**. However, the repulsive effect in Bi·$\overset{\text{C}}{\underset{\text{G12D}}{\text{K}}}\text{GDP}\cdot$S$\cdot \overset{\text{A}}{\underset{\text{G12D}}{\text{K}}}$ disrupts the interaction network among SW2, SOS1 and BI-3406.

**Fig 5:**
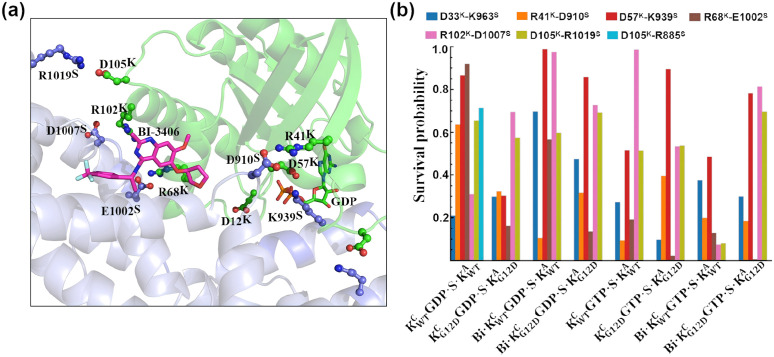
The salt bridges at the interface between KRas^C^ and SOS1 are analyzed. (a) The three-dimensional structure of salt bridges is shown. BI-3406 and the residues involved in salt bridge formation are highlighted by stick-ball representation. (b) The survival probability of salt bridges are statistically evaluated. For each salt bridge, the survival probability is defined as the cumulative time during which the distance between the charged groups of the interacting residues remained below 5 Å.

To evaluate the effect of the G12D mutation on electrostatic interactions at the KRas^C^–SOS1 interface, we calculated the survival probability of seven salt bridges (Fig 5). In $\overset{\text{C}}{\underset{\text{WT}}{\text{K}}}\text{GDP}\cdot$S$\cdot \overset{\text{A}}{\underset{\text{WT}}{\text{K}}}$, there are five salt bridges that have a survival probability longer than 0.5, which are R41^K^-D910^S^, D57^K^-K939^S^, R68^K^-E1002^S^, D105^K^-R1019^S^, and D105^K^-R885^S^. Among them, D105^K^-R885^S^ is observed only in $\overset{\text{C}}{\underset{\text{WT}}{\text{K}}}\text{GDP}\cdot$S$\cdot \overset{\text{A}}{\underset{\text{WT}}{\text{K}}}$. The G12D mutation significantly reduced the survival probability of salt Bridges. In $\overset{\text{C}}{\underset{\text{G12D}}{\text{K}}}\text{GDP}\cdot$S$\cdot \overset{\text{A}}{\underset{\text{G12D}}{\text{K}}}$, only R68^K^-E1002^S^ and D105^K^-R1019^S^ have survival probability longer than 0.5, while the survival probability of other salt bridges are all shorter than 0.3. The effect of BI-3406 on salt bridges is not significant, as five salt bridges still maintain survival probability longer than 0.5 in Bi·$\overset{\text{C}}{\underset{\text{WT}}{\text{K}}}\text{GDP}\cdot$S$\cdot \overset{\text{A}}{\underset{\text{WT}}{\text{K}}}$. A synergistic effect is observed from the G12D mutation and BI-3406 on salt bridges, with the strength of salt bridge in Bi·$\overset{\text{C}}{\underset{\text{WT}}{\text{K}}}\text{GDP}\cdot$S$\cdot \overset{\text{A}}{\underset{\text{WT}}{\text{K}}}$ is intermediate between that of $\overset{\text{C}}{\underset{\text{G12D}}{\text{K}}}\text{GDP}\cdot$S$\cdot \overset{\text{A}}{\underset{\text{G12D}}{\text{K}}}$ and Bi·$\overset{\text{C}}{\underset{\text{WT}}{\text{K}}}\text{GDP}\cdot$S$\cdot \overset{\text{A}}{\underset{\text{WT}}{\text{K}}}$. Furthermore, the charged residue D57^K^ is crucial for coordinating the Mg^2+^ ion (S5 Fig). The insertion of α-helix in SOS1 disrupts the interaction network between D57^K^ and Mg^2+^ ion. The stable D57^K^-K939^S^ salt bridge suggests that D57^K^ not only plays a vital role in coordinating the Mg^2+^ ion but also the interaction between SOS1 and KRas.

#### 2.3.2. G12D mutation and BI-3406 affect the interactions between KRas^C^GTP·Mg^2+^ and SOS1.

**Fig 6:**
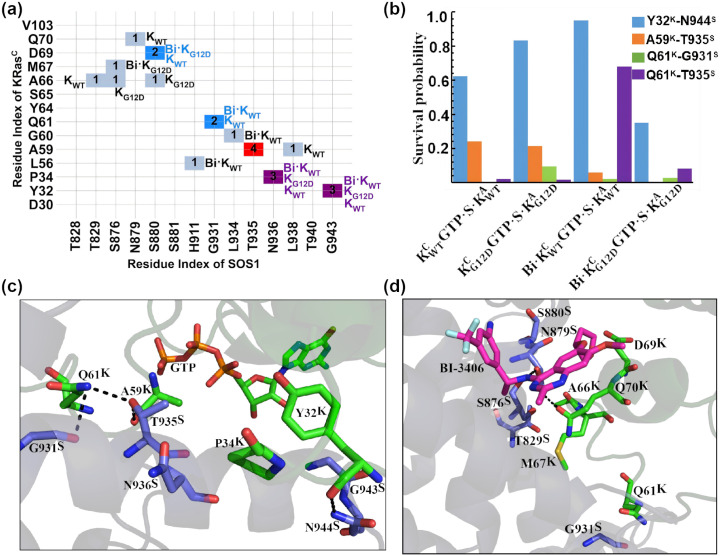
The overall interactions between KRas^C^GTP·Mg^2+^ and SOS1 are summarized. (a) The interaction matrix between KRas^C^ and SOS1 for the four KRas^C^GTP·Mg^2+^ ternary complexes is shown. K_WT_ represents the results of $\overset{\text{C}}{\underset{\text{WT}}{\text{K}}}\text{GTP}\cdot$S$\cdot \overset{\text{A}}{\underset{\text{WT}}{\text{K}}}$. K_G12D_ represents the results of $\overset{\text{C}}{\underset{\text{G12D}}{\text{K}}}\text{GTP}\cdot$S$\cdot \overset{\text{A}}{\underset{\text{G12D}}{\text{K}}}$. Bi·K_WT_ represent the results of Bi·$\overset{\text{C}}{\underset{\text{WT}}{\text{K}}}\text{GTP}\cdot$S$\cdot \overset{\text{A}}{\underset{\text{WT}}{\text{K}}}$. Bi·K_G12D_ represent the results of Bi·$\overset{\text{C}}{\underset{\text{G12D}}{\text{K}}}\text{GTP}\cdot$S$\cdot \overset{\text{A}}{\underset{\text{G12D}}{\text{K}}}$. The number ‘1’, ‘2’, ‘3’ and ‘4’ indicate that an interaction was observed in one, two, three and four of the simulated complexes, respectively. (b) The survival probability of key hydrogen bonds are shown as a bar graph. Here, the superscript “K” in Y32^K^, A59^K^ and Q61^K^ denotes residues from KRas^C^, while the superscript “S” in G931^S^, T935^S^ and N944^S^ denotes residues from SOS1. (c, d) the atomic interactions between KRas^C^ and SOS1, viewed from two distinct perspectives. The key residues are shown in stick-ball model. The dashed lines shows the hydrogen bonds.

Compared to KRas^C^GDP·Mg^2+^, the interactions in KRas^C^GTP·Mg^2+^ ternary complex are significantly weaker, particularly in SW2. In SW1, the hydrophobic effect among Y32^K^, P34^K^, N936^S^ and G943^S^ persists in K_WT_GTP·S·K_WT_, but is absent in Bi·$\overset{\text{C}}{\underset{\text{G12D}}{\text{K}}}\text{GTP}\cdot$S$\cdot \overset{\text{A}}{\underset{\text{G12D}}{\text{K}}}$ (Fig 6a). The Y32^K^-N944^S^ hydrogen bond persists for more than 600 ns in all KRas^C^GTP·Mg^2+^ ternary complex except Bi·$\overset{\text{C}}{\underset{\text{G12D}}{\text{K}}}\text{GTP}\cdot$S$\cdot \overset{\text{A}}{\underset{\text{G12D}}{\text{K}}}$. In Bi·$\overset{\text{C}}{\underset{\text{G12D}}{\text{K}}}\text{GTP}\cdot$S$\cdot \overset{\text{A}}{\underset{\text{G12D}}{\text{K}}}$, the dual influence from the G12D mutation and BI-3406 leads to a marked reduction in its survival probability to approximately 0.3 (Fig 6b and 6c).

In SW2, the hydrogen bond network among A59^K^, Q61^K^, G931^S^ and T935^S^ is broken in all KRas^C^GTP·Mg^2+^ ternary complex, with the exception of the hydrogen bond Q61^K^-T935^S^ in Bi·$\overset{\text{C}}{\underset{\text{WT}}{\text{K}}}\text{GTP}\cdot$S$\cdot \overset{\text{A}}{\underset{\text{WT}}{\text{K}}}$. In Bi·$\overset{\text{C}}{\underset{\text{WT}}{\text{K}}}\text{GTP}\cdot$S$\cdot \overset{\text{A}}{\underset{\text{WT}}{\text{K}}}$, Q61^K^-T935^S^ persists for over 600 ns (Fig 6b and 6c). The interaction between the N-terminal residues of SW2 and SOS1 is absent in all KRas^C^GTP·Mg^2+^ complexes, with the exception of A66^K^ and M67^K^ (Fig 6a). A66^K^ and M67^K^ remains proximate to T829^S^, S876^S^ and S880^S^ before BI-3406 binding. Additionally, the interaction between the C-terminal residues of SW2 and SOS1 is still observed, albeit with minor alterations. These changes further influence the binding model of BI-3406 described in **section 2.3.4**.

Compared to KRas^C^GDP·Mg^2+^ complex, the survival probability of salt bridges in all KRas^C^GTP·Mg^2+^ complex was significantly reduced (Fig 5). However, the D57^K^-K939^S^ salt bridge remains stable for over 400 ns across all KRas^C^GTP·Mg^2+^ ternary complexes. This observation reinforces the functional significance of D57^K^ in the interaction between KRas^C^ and SOS1. In addition, only salt bridges R102^K^-D1007^S^ and D105^K^-R1019^S^ exhibit survival probability longer than 0.4. Notably, BI-3406 significantly disrupts the salt bridges between KRas^C^GTP·Mg^2+^ and SOS1. In Bi·$\overset{\text{C}}{\underset{\text{WT}}{\text{K}}}\text{GTP}\cdot$S$\cdot \overset{\text{A}}{\underset{\text{WT}}{\text{K}}}$, all salt bridges except D57^K^-K939^S^ exhibit survival probability below 0.4. Combined with the results from KRas^C^GDP·Mg^2+^ complex, the conclusion is that the stability of the D57^K^-K939^S^ salt bridge is independent of the nucleotide molecule at binding pocket of KRas^C^. Overall, the interactions in KRas^C^GTP·Mg^2+^ are much weaker than that in KRas^C^GDP·Mg^2+^.

#### 2.3.4. G12D mutation attenuate the interactions between BI-3406 and KRas^C^:SOS1 complex.

**Fig 7:**
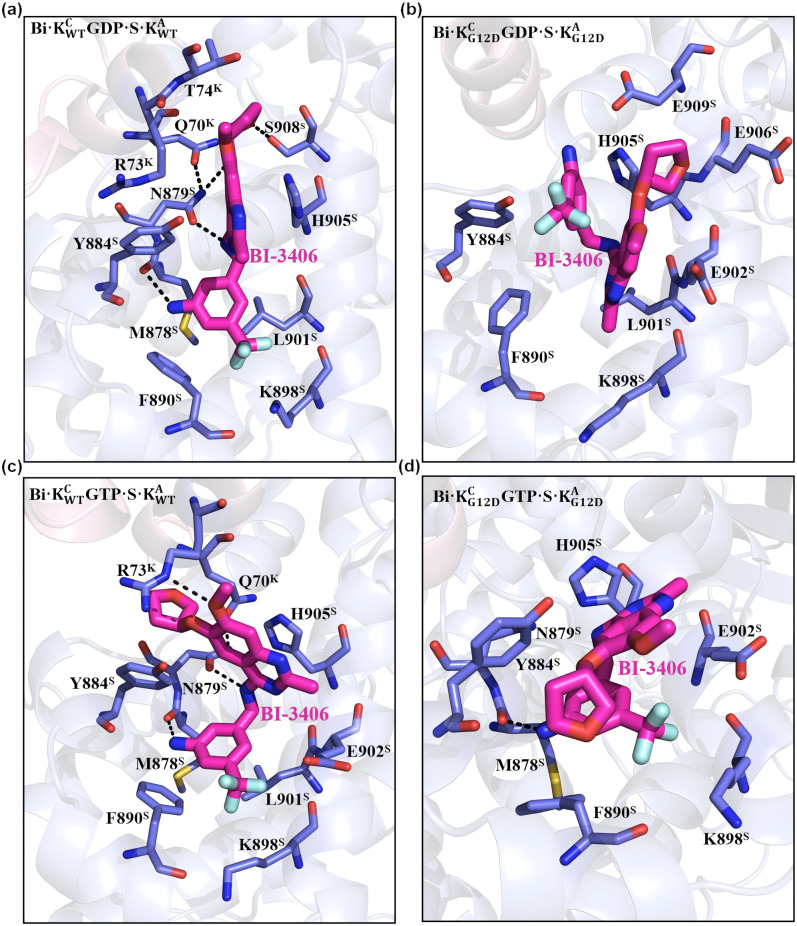
The overall interactions between BI-3406 and the ternary complex. The atomic interactions between BI-3406 and Bi·$\begin{array}{c} \overset{\text{C}}{\underset{\text{WT}}{\text{K}}}\text{GDP}\cdot \end{array}$S$\begin{array}{c} \cdot \overset{\text{A}}{\underset{\text{WT}}{\text{K}}} \end{array}$ (a), Bi·$\begin{array}{c} \overset{\text{C}}{\underset{\text{G12D}}{\text{K}}}\text{GDP}\cdot \end{array}$S$\begin{array}{c} \cdot \overset{\text{A}}{\underset{\text{G12D}}{\text{K}}} \end{array}$ (b), Bi·$\begin{array}{c} {{\text{K}}_{\text{\{WT}}}^{\text{C}}\text{GTP}\cdot \} \end{array}$S$\begin{array}{c} \cdot \overset{\text{A}}{\underset{\text{WT}}{\text{K}}} \end{array}$ (c), and Bi·$\begin{array}{c} \overset{\text{C}}{\underset{\text{G12D}}{\text{K}}}\text{GTP}\cdot \end{array}$S$\begin{array}{c} \cdot \overset{\text{A}}{\underset{\text{G12D}}{\text{K}}} \end{array}$ (d) ternary complex are illustrated.

As mentioned above, BI-3406 can regulate the interaction between SW2 and SOS1. The structural analysis reveals a hydrophobic effect between BI-3406 and the hydrophobic parts of Q70^K^, R73^K^ and T74^K^ in Bi·$\overset{\text{C}}{\underset{\text{WT}}{\text{K}}}\text{GDP}\cdot$S$\cdot \overset{\text{A}}{\underset{\text{WT}}{\text{K}}}$ (Fig 7a). Additionally, Q70^K^ forms a hydrogen bond with N879^S^, while N879^S^ simultaneously establishes two hydrogen bonds with BI-3406. Two of these three hydrogen bonds exhibit a probability greater than 0.6, indicating their stability (S8a Fig). A cation-π interactions is observed between the charged group of R73^K^ and Y884^S^, characterized by a distance that consistently remains below 5 Å. In addition, Y884^S^, together with H905^S^, sandwiches the quinazoline group of BI-3406 through robust π-π stacking interactions. The -NH2 and tetrahydrofuran groups of BI-3406 form hydrogen bond with M878^S^, with a probability approaching 0.6. The benzene ring in BI-3406 exhibits hydrophobic interactions with F890^S^ and L901^S^. Furthermore, the trifluoromethyl group participates in the electrostatic interactions with K898^S^, with a distance that fluctuates around 5 Å. Through the C-termini of SW2, BI-3406 can regulate the interaction between KRas^C^GDP·Mg^2+^ and SOS1.

In Bi·$\overset{\text{C}}{\underset{\text{WT}}{\text{K}}}\text{GTP}\cdot$S$\cdot \overset{\text{A}}{\underset{\text{WT}}{\text{K}}}$, the hydrophobic effect between SW2 and BI-3406 is weakened (Fig 7c). The intramolecular rotation of BI-3406 results in a different orientation of the its quinazoline group (Figs 7c and S9). However, the π-π stacking interaction among H905^S^, Y884^S^ and the quinazoline group of BI-3406 persist (S8a Fig). Meanwhile, the cation-π interaction between R73^K^ and Y884^S^ weakens, as reflected by an increased distance (to 6 Å) between the charged group of R73^K^ and Y884^S^. In Bi·$\overset{\text{C}}{\underset{\text{WT}}{\text{K}}}\text{GTP}\cdot$S$\cdot \overset{\text{A}}{\underset{\text{WT}}{\text{K}}}$, three hydrogen bonds, Q70^K^-N879^S^, Bi-N879^S^ and Bi-M878^S^, remain stable, each with a probability greater than 0.6. Furthermore, the binding model of the trifluoromethyl group in BI-3406 is maintained, as indicated by a distance converging to approximately 5 Å, consistent with that in Bi·$\overset{\text{C}}{\underset{\text{WT}}{\text{K}}}\text{GDP}\cdot$S$\cdot \overset{\text{A}}{\underset{\text{WT}}{\text{K}}}$.

The structural analysis reveals that BI-3406 penetrates deeper into the binding pocket of SOS1 in the G12D mutated complexes, irrespective of whether the complex is Bi·$\overset{\text{C}}{\underset{\text{G12D}}{\text{K}}}\text{GDP}\cdot$S$\cdot \overset{\text{A}}{\underset{\text{G12D}}{\text{K}}}$ or Bi·$\overset{\text{C}}{\underset{\text{G12D}}{\text{K}}}\text{GTP}\cdot$S$\cdot \overset{\text{A}}{\underset{\text{G12D}}{\text{K}}}$(Fig 7b and 7d). The distance between the geometrical center of the quinazoline group in BI-3406 and the geometrical center of CA atoms from F890^S^ and K898^S^ converges to approximately 7 Å and 9 Å in Bi·$\overset{\text{C}}{\underset{\text{G12D}}{\text{K}}}\text{GDP}\cdot$S$\cdot \overset{\text{A}}{\underset{\text{G12D}}{\text{K}}}$ and Bi·$\overset{\text{C}}{\underset{\text{G12D}}{\text{K}}}\text{GTP}\cdot$S$\cdot \overset{\text{A}}{\underset{\text{G12D}}{\text{K}}}$, respectively (S8 Fig). In contrast, in Bi·$\overset{\text{C}}{\underset{\text{WT}}{\text{K}}}\text{GDP}\cdot$S$\cdot \overset{\text{A}}{\underset{\text{WT}}{\text{K}}}$ and Bi·$\overset{\text{C}}{\underset{\text{WT}}{\text{K}}}\text{GTP}\cdot$S$\cdot \overset{\text{A}}{\underset{\text{WT}}{\text{K}}}$, the distance consistently stabilizes at ~12 Å. The movement of BI-3406 results in the disruption of the interactions between the C-termini of SW2 and SOS1. In Bi·$\overset{\text{C}}{\underset{\text{G12D}}{\text{K}}}\text{GDP}\cdot$S$\cdot \overset{\text{A}}{\underset{\text{G12D}}{\text{K}}}$, there is no hydrogen bond between BI-3406 and SOS1 (S8 Fig). The electrostatic interaction between the trifluoromethyl group and K898^S^ is also severed, with a distance closing to 12 Å. H905^S^ and Y884^S^ exhibit π-π stacking interactions with the benzene ring of BI-3406. In Bi·$\overset{\text{C}}{\underset{\text{G12D}}{\text{K}}}\text{GTP}\cdot$S$\cdot \overset{\text{A}}{\underset{\text{G12D}}{\text{K}}}$, only the hydrogen bond between -NH2 linked to the tetrahydrofuran group and M878^S^ is maintained. Moreover, the cation-π interaction between R73^K^ and Y884^S^, as well as the electrostatic interaction between the trifluoromethyl group and K898^S^, persists, with distances similar to those observed in WT complex. In summary, compared to the interactions in WT KRas^C^ ternary complexes, the interactions between BI-3406 and the G12D mutated ternary complexes are significantly weaker.

#### 2.3.5. G12D mutation and BI-3406 reduce the binding affinity of KRas^C^ for SOS1.

**Fig 8:**
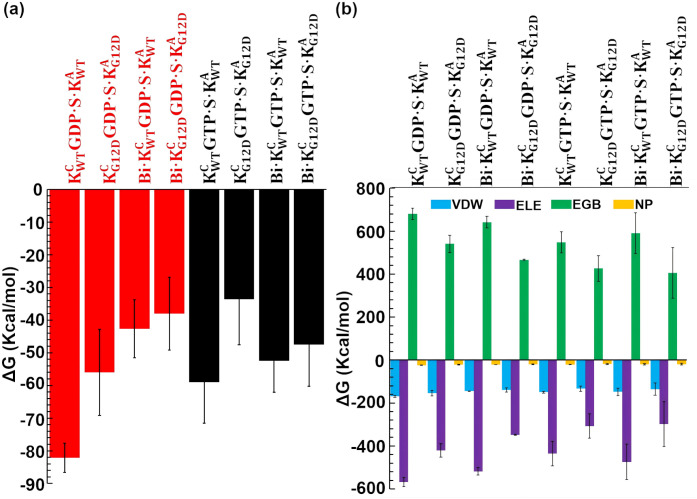
The binding affinity between KRas^C^ and SOS1 are calculated. (a) The binding affinity with standard deviation between KRas^C^ and SOS1 for each ternary complex is calculated from three independent trajectories. (b) The binding affinity was decomposed into contributions from van der Waals (VDW), electrostatic (ELE), nonpolar solvation (NP), and polar solvation (EGB) interactions.

The binding affinity between KRas^C^ and SOS1 is calculated using the MM/GBSA module within AMBER package, with calculations performed over three independent trajectories. The results indicate that WT KRas^C^GDP·Mg^2+^ exhibits the strongest binding affinity, with a value as high as -82.2 kcal/mol in $\overset{\text{C}}{\underset{\text{WT}}{\text{K}}}\text{GDP}\cdot$S$\cdot \overset{\text{A}}{\underset{\text{WT}}{\text{K}}}$ (Fig 8a). Introduction of the G12D mutation reduces the affinity to -56.0 kcal/mol in $\overset{\text{C}}{\underset{\text{G12D}}{\text{K}}}\text{GDP}\cdot$S$\cdot \overset{\text{A}}{\underset{\text{G12D}}{\text{K}}}$, while BI-3406 binding alone decreases it to -42.7 kcal/mol in Bi·$\overset{\text{C}}{\underset{\text{WT}}{\text{K}}}\text{GDP}\cdot$S$\cdot \overset{\text{A}}{\underset{\text{WT}}{\text{K}}}$. The simultaneous presence of both the G12D mutation and BI-3406 in Bi·$\overset{\text{C}}{\underset{\text{G12D}}{\text{K}}}\text{GDP}\cdot$S$\cdot \overset{\text{A}}{\underset{\text{G12D}}{\text{K}}}$ results in a diminished affinity of -38.0 kcal/mol. The ΔΔG values reveal that the G12D mutation causes a decrease in binding affinity of 26.2 kcal/mol. BI-3406 results in a reduction of 39.5 kcal/mol. The G12D mutation and BI-3406 exhibit a slight synergistic effect, resulting in a 44.1 kcal/mol reduction in the binding free energy between KRas and SOS1. (S10 Fig).

Compared to KRas^C^GDP·Mg^2+^ complex, the binding affinity of KRas^C^GTP·Mg^2+^ is significantly reduced. The binding affinity is -59.0 kcal/mol in $\overset{\text{C}}{\underset{\text{WT}}{\text{K}}}\text{GTP}\cdot$S$\cdot \overset{\text{A}}{\underset{\text{WT}}{\text{K}}}$ and -33.6 kcal/mol in $\overset{\text{C}}{\underset{\text{G12D}}{\text{K}}}\text{GTP}\cdot$S$\cdot \overset{\text{A}}{\underset{\text{G12D}}{\text{K}}}$, respectively. The results indicate that KRas^C^GDP·Mg^2+^ has a stronger binding affinity for BI-3406-free SOS1 compared to KRas^C^GTP·Mg^2+^. Interestingly, BI-3406 has minimal effect on the binding of KRas^C^GTP·Mg^2+^ to SOS1, with a binding affinity of -52.5 kcal/mol in Bi·$\overset{\text{C}}{\underset{\text{WT}}{\text{K}}}\text{GTP}\cdot$S$\cdot \overset{\text{A}}{\underset{\text{WT}}{\text{K}}}$. In Bi·$\overset{\text{C}}{\underset{\text{G12D}}{\text{K}}}\text{GTP}\cdot$S$\cdot \overset{\text{A}}{\underset{\text{G12D}}{\text{K}}}$, the binding affinity decreases to -47.5 kcal/mol. The ΔΔG shows that BI-3406 results in a reduction in binding affinity of 6.5 kcal/mol in WT KRas^C^GTP·Mg^2+^ complex which is notably smaller than the corresponding descrease of 39.5 kcal/mol observed for WT KRas^C^GDP·Mg^2+^ complex (S10 Fig). However, the G12D mutation leads to a decrease in the binding affinity of 25.3 kcal/mol in KRas^C^GTP·Mg^2+^, a value comparable to the corresponding reduction of 26.2 kcal/mol observed for KRas^C^GDP·Mg^2+^. By comparing with the KRas^C^GDP·Mg^2+^ complex, the G12D mutation reduces the binding affinity of both KRas^C^GDP·Mg^2+^ and KRasGTP·Mg^2+^ to SOS1, but does not alter the relative affinity between the two states (23.2 kcal/mol for KRas^C^GDP·Mg^2+^ and 22.4 for KRas^C^GTP·Mg^2+^). At the same time, G12D mutation accelerates the rate of GDP extraction [67], thereby leading to tumors. However, BI-3406 selectively attenuates the binding affinity of WT KRasGDP·Mg^2+^ and slightly affects the binding affinity of WT KRasGTP·Mg^2+^. In results, BI-3406 increase binding affinity of G12D-mutant KRasGTP·Mg^2+^, thereby making the catalytic binding pocket of SOS1 prefers to binding KRasGTP·Mg^2+^, inhibiting the GDP-to-GTP exchange and inhibiting tumor growth.

The binding affinity is further decomposed into Δ*E*_VDW_, Δ*E*_ELE_, Δ*G_GB_* and Δ*G_NP_*, as described in eq.(1). For each ternary complex, all Δ*E*_VDW_ values (the hydrophobic contribution in gas phase) are about -150 kcal/mol with fluctuations less than 10 kcal/mol (Fig 8b). All Δ*G_NP_* values (the hydrophobic contribution from solvation) are about -20 kcal/mol with fluctuations less than 2 kcal/mol (Fig 8b). The results indicate that the G12D mutation and BI-3406 have minimal impact on the hydrophobic contribution of KRas^C^ binding to SOS1. The electrostatic interactions represented by Δ*E*_ELE_ and Δ*G*_GB_ are significantly influenced by the G12D mutation. In $\overset{\text{C}}{\underset{\text{WT}}{\text{K}}}\text{GDP}\cdot$S$\cdot \overset{\text{A}}{\underset{\text{WT}}{\text{K}}}$, the Δ*E*_ELE_ value is the highest at -568.3 kcal/mol (Fig 8b). The G12D mutation causes the Δ*E*_ELE_ value decreasing to -421.3 kcal/mol in $\overset{\text{C}}{\underset{\text{G12D}}{\text{K}}}\text{GDP}\cdot$S$\cdot \overset{\text{A}}{\underset{\text{G12D}}{\text{K}}}$. In Bi·$\overset{\text{C}}{\underset{\text{WT}}{\text{K}}}\text{GDP}\cdot$S$\cdot \overset{\text{A}}{\underset{\text{WT}}{\text{K}}}$, the Δ*E*_ELE_ value is -518.4 kcal/mol. While in Bi·$\overset{\text{C}}{\underset{\text{G12D}}{\text{K}}}\text{GDP}\cdot$S$\cdot \overset{\text{A}}{\underset{\text{G12D}}{\text{K}}}$, the Δ*E*_ELE_ value decreases to -348.1 kcal/mol. The results indicate that the G12D mutation heavily affect the electrostatic interactions in KRas^C^GDP·Mg^2+^. The Δ*G*_GB_ value exhibits a similar variation trend as the Δ*E*_ELE_ value. The G12D mutation leads to a significant reduction of the Δ*G*_GB_ value in KRas^C^GDP·Mg^2+^ (Fig 8b). In KRas^C^GTP·Mg^2+^, the electrostatic contribution is marginally smaller than in KRas^C^GDP·Mg^2+^. In $\overset{\text{C}}{\underset{\text{WT}}{\text{K}}}\text{GTP}\cdot$S$\cdot \overset{\text{A}}{\underset{\text{WT}}{\text{K}}}$, the Δ*E*_ELE_ and Δ*G*_GB_ values are -435.7 kcal/mol and 547.5 kcal/mol, respectively. BI-3406 raises the Δ*E*_ELE_ value to -474.3 kcal/mol and lowers the Δ*G*_GB_ value to 590.6 kcal/mol in Bi·$\overset{\text{C}}{\underset{\text{WT}}{\text{K}}}\text{GTP}\cdot$S$\cdot \overset{\text{A}}{\underset{\text{WT}}{\text{K}}}$. However, the G12D mutation increases the Δ*E*_ELE_ value to -307.4 kcal/mol and decreases the Δ*G*_GB_ value to -426.3 kcal/mol in $\overset{\text{C}}{\underset{\text{G12D}}{\text{K}}}\text{GTP}\cdot$S$\cdot \overset{\text{A}}{\underset{\text{G12D}}{\text{K}}}$. In Bi·$\overset{\text{C}}{\underset{\text{G12D}}{\text{K}}}\text{GTP}\cdot$S$\cdot \overset{\text{A}}{\underset{\text{G12D}}{\text{K}}}$, the Δ*E*_ELE_ and Δ*E*_GB_ values are -298.6 kcal/mol and 406.0 kcal/mol, respectively. The results shows that the G12D mutation also heavily affect the electrostatic contribution in KRas^C^GTP·Mg^2+^.

### 2.6. The G12D mutation alters the electrostatic potential of the KRas-SOS1 interface

**Fig 9:**
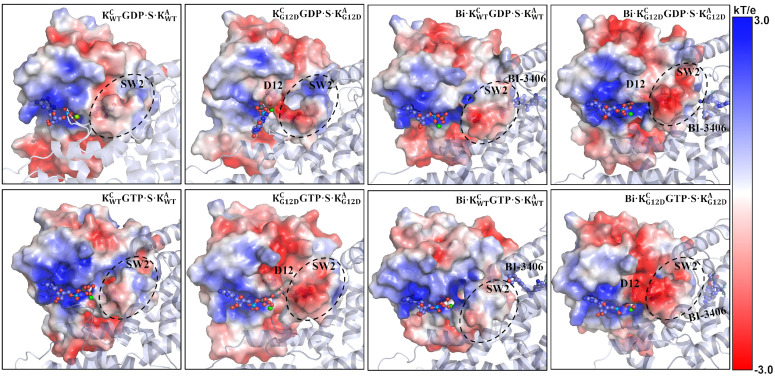
The electrostatic potential energy surface of KRas^C^ in the representative structure. The potentials are on a red-white-blue [±3 kT/e] color map. The blue is electropositive and red is electronegative. The SW2 region is highlighted by a dashed circle.

The electrostatic potential (ESP) of KRas^C^ is graphically display in Fig 9. The binding pocket of the nucleotide molecule is in an obviously positive electrostatic state. The negatively charged D12 is nearby the binding pocket and offset some of the positive charge effects, whether in KRas^C^GDP·Mg^2+^ or KRas^C^GTP·Mg^2+^ complexes. The offsetting effect in KRas^C^GTP·Mg^2+^ is more pronounced. The SW2 region is rendered in white based on ESP, indicating a neutral electrostatic potential in WT KRas^C^GDP·Mg^2+^ and KRas^C^GTP·Mg^2+^. The introduction of the negatively charged D12 also affects the ESP of SW2, as highlighted by the dotted-line circle in Fig 9. In the G12D mutated KRas^C^ complexes, the ESP of SW2 is colored in red, which indicates a negatively charged electrostatic state. Particularly at the BI-3406 bound state, SW2 exhibits a stronger negative electrostatic character, colored in deep red. The results indicate although BI-3406 is far away from SW2, the conformation and electrostatic state of SW2 still affect BI-3406 binding. S1 Fig indicates that SW2 plays a key role for inhibitors targeting the G12D-mutant KRas. Therefore, when designing inhibitors against G12D-mutant KRas, the changes of the electrostatic environment caused by the G12D mutation must be considered. The negative electrostatic environment induced by the G12D mutation highlightes the potential of the positively charged molecules as promising drug candidates.

## 3. Conclusion

The inactive KRasGDP·Mg^2+^ state can be converted back to the active KRasGTP·Mg^2+^ state with the assistance of SOS1. SOS1 has the catalytic binding site and allosteric binding sites for KRas, leading to the functional (KRas^C^GDP·Mg^2+^)·SOS1·(KRas^A^GTP·Mg^2+^) ternary complex. Currently, there is no experimental structure of the ternary complex. Our previous work observed the special structure of KRasGDP·Mg^2+^_S1.2_ binding with SOS1 [54]. Based on the structure, we construct the ternary complex that remains stable throughout a 1-μs MD simulation, enabling us to investigate how the G12D mutation and the SOS1 inhibitor BI-3406 modulate the KRas^C^-SOS1 interaction.

The findings indicate that SW1 and SW2 regions are crucial not only for stabilizing the special structure of KRas but also for binding of SOS1. The G12D mutation introduces a net negative charge that repels E62, E63 of SW2, reducing the binding affinity by approximately by ~ 26.2 kcal/mol for KRas^C^GDP·Mg^2+^ and ~ 25.3 kcal/mol for KRas^C^GTP·Mg^2+^, without altering their relative affinity. The G12D mutation is also found to increase GDP fluctuation within the binding pocket, consistent with Hu et al.’s observation that G12D accelerates GDP extraction, thereby increasing active KRasGTP probability and promoting tumor growth. BI-3406, known to inhibit tumor growth by disrupting KRas^C^GDP·Mg^2+^ and SOS1, was found here to selectively attenuate the binding affinity of WT KRasGDP·Mg^2+^, while only mildly affecting the binding affinity of WT KRasGTP·Mg^2+^. Interestingly, BI-3406 enhances the binding affinity of G12D-mutant KRasGTP·Mg^2+^, shifting the preference of the SOS1 catalytic pocket toward KRasGTP·Mg^2 +^ . This shift disrupts the GDP-to-GTP exchange and suppresses tumor progression. Structural analysis reveals that BI-3406 forms a hydrogen bond network with residues Q70^K^ and N879^S^, which in turn modulates the interaction between SW2 and SOS1. In summary, this study elucidates the molecular mechanism by which the G12D mutation and BI-3406 modulate the interaction between KRas and SOS1, providing insights into KRas-driven cancers.

## 4. Materials and methods

### 4.1. System setup

The experimental structure PDB ID 7KFZ [58] was used for constructing the initial complex. Residues Q566-N1044 encompassing both CDC25 and REM domains of SOS1 were retained for simulation. In each complex, KRasGTP·Mg^2+^ consistently occupies the allosteric site (designated by the superscripted KRas^A^), the superscripted KRas^C^GDP·Mg^2+^ or KRas^C^GTP·Mg^2+^ bound at the catalytic site was labeled KRas^C^. Missing residues were modeled using the MODELLER webserver [74]. Initial coordinates for KRas^C^GDP·Mg^2+^ or KRas^C^GTP·Mg^2+^ derive from aligned PDB ID 4OBE [75] and 3GFT [54], respectively, superimposed onto KRas^C^ in PDB ID 7KFZ. Initial coordinates of SOS1 and KRas^A^GTP·Mg^2+^ were kept from PDB ID 7KFZ, with GNP systematically reverted to GTP. To investigate the effect of SOS1 inhibitor BI-3406 on the interactions, initial coordinates of BI-3406 extracted from aligned PDB ID 6SCM [34] onto PDB ID 7KFZ, then merged seamlessly into the ternary complex. Given BI-3406’s reported efficacy against G12D KRas mutant, the residue Gly12 of KRas was mutated into Asp12 in each ternary complex. Collectively, eight systems—detailed in S1 Table —were simulated for three independent runs.

### 4.2. Details of MD simulation

The constructed systems were fed into AMBER22 package [76] to perform MD simulation. The Amber ff19SB [77] force files were selected for proteins. The force fields of GTP and GDP were directly adopted from our previous work [78–80]. The parameters of BI-3406 were prepared using the GAFF2 [81] force field. Atomic charges of BI-3406 were fitted through the RESP [82] methodology, derived from ESP calculated in Gaussian09 [83] software using the HF/6-31G* basis set [84,85]. The whole system was solvated in a 10 Å truncated octahedron box filled with TIP3P water molecules. The counterions were added for neutralizing the system. The protonated states of KRas and SOS1 were set to the states at physiological pH, where residues LYS and ARG have a unit positive charge, residues GLU and ASP have a unit negative charge, and the protonation state of residues HIS were set to the default HIE in LEAP module.

Each MD simulation has four steps: minimization, heating, relaxation, and production. In minimization, the system firstly undergoes 50,000 minimization steps with proteins harmonically restrained (force constant: 100 kcal/mol/Å^2^). Subsequently, minimization proceeds unrestrained until the energy converges to within 10^-4^ kcal·mol^-1^·Å^-1^. During MD simulation, hydrogen-involving bonds were constrained through the SHAKE [86] algorithm. The integration time step was set to 2 fs. The cutoffs of both van der Waals (VDW) and real-space electrostatic interaction were 10 Å. Long-range electrostatic interactions were treated through the Particle Mesh Ewald (PME) [87] method. System temperature was controlled via the Langevin Dynamics [88] approach, with collision frequency stabilized at 2.0 ps and environmental pressure maintained at 1 atm. During thermal equilibration, temperatures ascended gradually from 0 K to 300 K over 1 ns within the NVT ensemble, while protein backbone atoms was constrained by 50 kcal/mol/Å^2^ harmonic potentials. Subsequent system relaxation occurred at three stages within the NPT ensemble, sequentially easing restraints from 25 to 10 kcal/mol/Å^2^ and ultimately 0 kcal/mol/Å². The production was performed for 1000 ns without restraints in the NPT ensemble. The atomic coordinates were collected at an interval of every 2 ps.

### 4.3. Details of MM/GBSA calculation

The binding free energy between KRas^C^ and SOS1 was calculated utilizing the molecular mechanics/generalized Born surface area (MM/GBSA) methodology [89]. In MM/GBSA, the binding affinity was calculated according to eq.(1) below:


$$\Delta G=\Delta H-T\Delta S=\Delta E_{ELE}+\Delta E_{VDW}+\Delta G_{GB}+\Delta G_{NP}-T\Delta S$$ (1)

Eq.(1) indicates that the energy comprises enthalpic and entropic contribution. Based on the experience, neglecting the contribution of entropy is feasible. Enthalpy is further decomposed into four terms: *ΔE*_ELE_, *ΔE*_VDW_, *ΔG*_GB_ and *ΔG*_NP_. The *ΔE*_ELE_ and *ΔE*_VDW_ is the contribution of electrostatic and VDW interactions in gas phase, while *ΔG*_GB_ and *ΔG*_NP_ represent the polar and nonpolar contribution of solvation free energy. The *ΔE*_ELE_ and *ΔE*_VDW_ were calculated in sander module of AMBER package. The analytic generalized Born (GB) [90,91] method was applied to calculate *ΔG*_GB_, whereas *ΔG*_NP_ derives from its approximate dependence on protein surface area. The protein surface area is estimated via the linear combination of pairwise overlaps (LCPO) [92] method. One hundred snapshots were evenly extracted from the last 200-ns trajectory for the MM/GBSA analysis. During the MM/GBSA calculation, KRas^C^GDP·Mg^2+^ or KRas^C^GTP·Mg^2+^ are defined as ligand. SOS1 is set to receptor. The generalized born term is set to 5 (igb = 5), and the salt ionic concentration is set to 0.15 M. Other parameters remain at default. We also tested the results with the igb8 term, and the energy trends are consistent with those from igb5 (S11 Fig).

### 4.4. Trajectory analysis

The cpptraj [93] module and in-house code were employed to analyze the trajectories. Root mean square deviation (RMSD) values were calculated using the initial structure as reference. The cpptraj was also applied to calculate the atomic distance. The in-house python code derives the averaged RMSD and distance values with their standard deviations for the last 200-ns trajectory. All visualizations were generated using in-house Python plotting routines. Representative structures were identified through averagelinkage clustering analysis applied to the last 200-ns trajectory. Molecular graphics were rendered using PyMOL [94] and Chimera [95] software. Salt bridges were identified by monitoring geometric center distances between charged groups (-NH3, -COO), while hydrogen bonds required the donor-acceptor distance ≤ 3.5 Å and donor-H-acceptor angle ≥ 120°. The survival probability of each hydrogen bond is calculated as the number of frames in which it is formed divided by the total number of frames. Electrostatic potential landscapes, computed via the APBS [96] method, were projected onto protein surfaces using PyMOL’s volumetric rendering capabilities.

## Supporting information

S1 Table Summarizes the simulated systems with abbreviated name. (DOCX)

S1 Fig Represents the experimental structures of KRas in complex with inhibitors. (DOCX)

S2 Fig Shows the RMSD values of complex function as simulation time. (DOCX)

S3 Fig Shows the 2D-FEL and 2D-RMSD values of KRas^C^. (DOCX)

S4 Fig Is the representative structures of KRas^C^. (DOCX)

S5 Fig Shows the six-coordination of Mg2 + ion. (DOCX)

S6 Fig Describes the RMSD values and structures of GDP/GTP within KRas^C^. (DOCX)

S7 Fig Represents the distance between G12/D12 and E62. (DOCX)

S8 Fig Shows the hydrogen bonds and distances between BI-3406 and complex. (DOCX)

S9 Fig Shows the dihedral angle (N1-C7-C15-N3) distribution within BI-3406. (DOCX)

S10 Fig Calculates the relative binding free energy between KRas^C^ and SOSl. (DOCX)

S11 Fig Calculates the binding affinity from igb = 5 and igb = 8 terms. (DOCX)
