# Supplementary material for: Computational insights on the molecular interplay between KRas (G12D mutation) and SOS1 modulated by the inhibitor BI-3406
Source: PLoS Comput Biol. 2026 Apr 29;22(4):e1014213. doi: 10.1371/journal.pcbi.1014213 (PMC13155684; doi:10.1371/journal.pcbi.1014213)
Supplement: S1 Table — (DOCX) [file pcbi.1014213.s001.docx]

**S1 Table.** All simulated systems in this work. There are 8 systems in total. The first column lists the simulated systems. The second column shows the abbreviated names of systems. The third column gives the simulated time of each system.

| **Systems** | **Abbreviations** | **Times** |
| --- | --- | --- |
| ($\mathrm{KRas}_{\mathrm{WT}}^{C}$GDP·Mg^2+^)·SOS1·($\mathrm{KRas}_{\mathrm{WT}}^{A}$GTP·Mg^2+^) | $K_{\mathrm{WT}}^{C}$GDP·S·$K_{\mathrm{WT}}^{A}$ | 1μs |
| ($\mathrm{KRas}_{G12D}^{C}$GDP·Mg^2+^)·SOS1·($\mathrm{KRas}_{G12D}^{A}$GTP·Mg^2+^) | $K_{G12D}^{C}$GDP·S·$K_{G12D}^{A}$ | 1μs |
| BI-3406·($\mathrm{KRas}_{\mathrm{WT}}^{C}$GDP·Mg^2+^)·SOS1·($\mathrm{KRas}_{\mathrm{WT}}^{A}$GTP·Mg^2+^) | Bi·$K_{\mathrm{WT}}^{C}$GDP·S·$K_{\mathrm{WT}}^{A}$ | 1μs |
| BI-3406·($\mathrm{KRas}_{G12D}^{C}$GDP·Mg^2+^)·SOS1·($\mathrm{KRas}_{G12D}^{A}$GTP·Mg^2+^) | Bi·$K_{G12D}^{C}$GDP·S·$K_{G12D}^{A}$ | 1μs |
| ($\mathrm{KRas}_{\mathrm{WT}}^{C}$GTP·Mg^2+^)·SOS1·($\mathrm{KRas}_{\mathrm{WT}}^{A}$GTP·Mg^2+^) | $K_{\mathrm{WT}}^{C}$GTP·S·$K_{\mathrm{WT}}^{A}$ | 1μs |
| ($\mathrm{KRas}_{G12D}^{C}$GTP·Mg^2+^)·SOS1·($\mathrm{KRas}_{G12D}^{A}$GTP·Mg^2+^) | $K_{G12D}^{C}$GTP·S·$K_{G12D}^{A}$ | 1μs |
| BI-3406·($\mathrm{KRas}_{\mathrm{WT}}^{C}$GTP·Mg^2+^)·SOS1·($\mathrm{KRas}_{\mathrm{WT}}^{A}$GTP·Mg^2+^) | Bi·$K_{\mathrm{WT}}^{C}$GTP·S·$K_{\mathrm{WT}}^{A}$ | 1μs |
| BI-3406·($\mathrm{KRas}_{G12D}^{C}$GTP·Mg^2+^)·SOS1·($\mathrm{KRas}_{G12D}^{A}$GTP·Mg^2+^) | Bi·$K_{G12D}^{C}$GTP·S·$K_{G12D}^{A}$ | 1μs |
