# Supplementary material for: Computational insights on the molecular interplay between KRas (G12D mutation) and SOS1 modulated by the inhibitor BI-3406
Source: PLoS Comput Biol. 2026 Apr 29;22(4):e1014213. doi: 10.1371/journal.pcbi.1014213 (PMC13155684; doi:10.1371/journal.pcbi.1014213)

**S1 Fig.** The experimental structures of the active KRas (PDB ID: 3GFT), sotorasib-bound G12C KRas mutant (PDB ID: 6OIM), adagrasib-bound G12C KRas mutant (PDB ID: 6USX) and MRTX1133-bound G12D KRas mutant (PDB ID: 7T47).


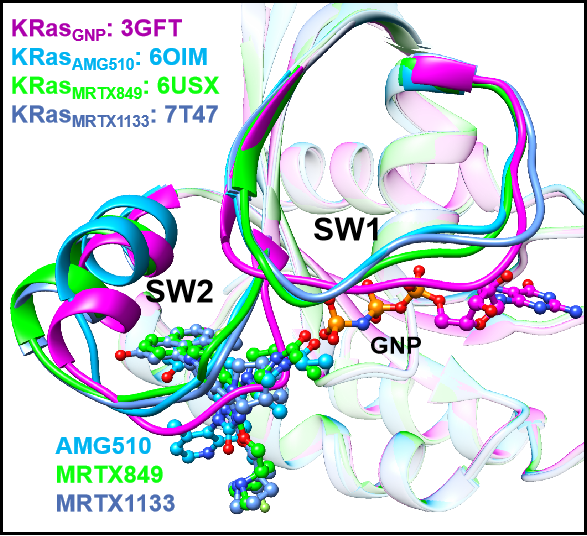

Supplement: S1 Fig — (DOCX) [file pcbi.1014213.s002.docx]
