# Supplementary material for: Computational insights on the molecular interplay between KRas (G12D mutation) and SOS1 modulated by the inhibitor BI-3406
Source: PLoS Comput Biol. 2026 Apr 29;22(4):e1014213. doi: 10.1371/journal.pcbi.1014213 (PMC13155684; doi:10.1371/journal.pcbi.1014213)

**S2 Fig.** The heavy-atom RMSD values for each ternary complex from three independent trajectories evolve as the simulation time.


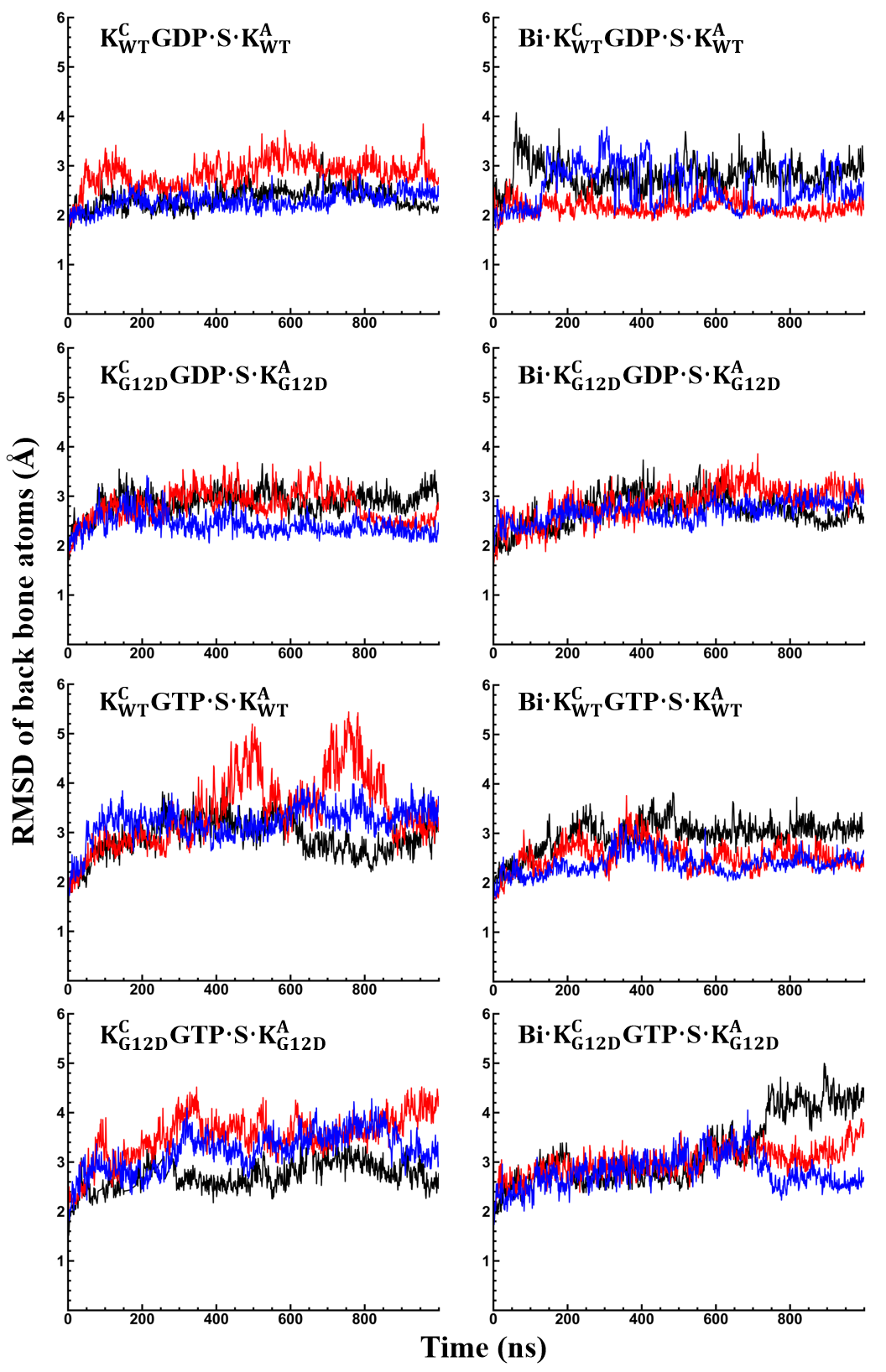

Supplement: S2 Fig — (DOCX) [file pcbi.1014213.s003.docx]
