# Supplementary material for: Computational insights on the molecular interplay between KRas (G12D mutation) and SOS1 modulated by the inhibitor BI-3406
Source: PLoS Comput Biol. 2026 Apr 29;22(4):e1014213. doi: 10.1371/journal.pcbi.1014213 (PMC13155684; doi:10.1371/journal.pcbi.1014213)

**S3 Fig.** (a) The representative KRas^C^ structures from the ternary complexes are projected onto the 2D-FEL of KRasGDP·Mg^2+^ from our previous REMD simulation. The dashed rectangle outlines the projection region of seven representative KRas^C^ structures. The structure of Bi·$K_{G12D}^{C}$GTP·S·$K_{G12D}^{A}$ far away from the region is highlighted. (b) The two dimensional RMSD (2D-RMSD) values of PDB ID 3GFT and the representative KRas^C^ structures in the ternary complexes. The upper matrix is the 2D-RMSD values of back bone atoms of SW1 region. The lower matrix is the 2D-RMSD values of back bone atoms of KRas^C^.


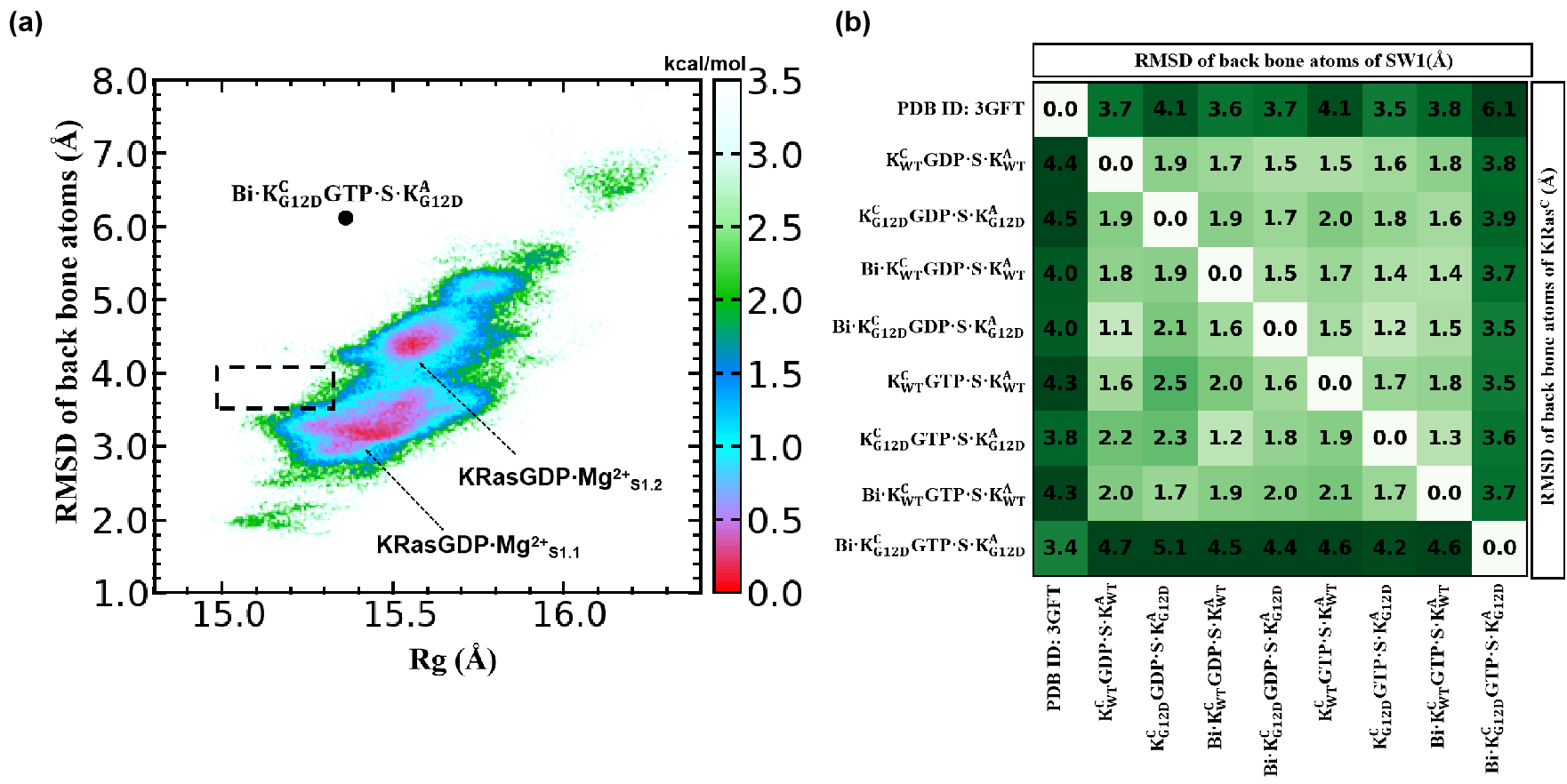

Supplement: S3 Fig — (DOCX) [file pcbi.1014213.s004.docx]
