# Supplementary material for: Computational insights on the molecular interplay between KRas (G12D mutation) and SOS1 modulated by the inhibitor BI-3406
Source: PLoS Comput Biol. 2026 Apr 29;22(4):e1014213. doi: 10.1371/journal.pcbi.1014213 (PMC13155684; doi:10.1371/journal.pcbi.1014213)

**Supporting Information**

**Computational Insights on the Molecular Interplay Between KRas (G12D Mutation) and SOS1 Modulated by the Inhibitor BI-3406**

Juan Zeng,^1,^ * YiXuan Lan,^1^ and Fei Xia^2, 3^

^1^*School of Biomedical Engineering, Guangdong Medical University, Dongguan 523808, China.*

^2^*Shanghai Engineering Research Center of Molecular Therapeutics and New Drug Development, School of Chemistry and Molecular Engineering, East China Normal University, Shanghai 200062, China.*

*^3^NYU-ECNU Center for Computational Chemistry at NYU Shanghai, Shanghai 200062, China.*

**Corresponding Authors:** [azengjuan@gdmu.edu.cn](mailto:azengjuan@gdmu.edu.cn) (Juan Zeng)

**Table S1:** The simulated systems with abbreviated name in this work.

**Figure S1:** the experimental structures of KRas with some inhibitors.

**Figure S2:** The RMSD values of complex function as simulation time.

**Figure S3:** The FEL and 2D-RMSD values of KRas^C^.

**Figure S4:** The representative structures of KRas^C^.

**Figure S5:** The six-coordination of Mg2+ ion.

**Figure S6:** The RMSD values and structures of GDP/GTP within KRas^C^.

**Figure S7:** The distance between G12/D12 and E62.

**Figure S8:** The hydrogen bonds and distances between BI-3406 and complex.

**Figure S9:** The distribution of the dihedral angle N1-C7-C15-N3 within BI-3406.

**Figure S10:** The relative binding free energy (∆∆G) between KRas^C^ and SOSl.

**Figure S11:** The binding affinity from igb=5 and igb=8 terms.

**S4 Fig.** (a) The representative structures from KRas^C^GDP·Mg^2+^ ternary complexes are viewed in Chimera. (b) The representative structures from KRas^C^GTP·Mg^2+^ ternary complexes are shown.


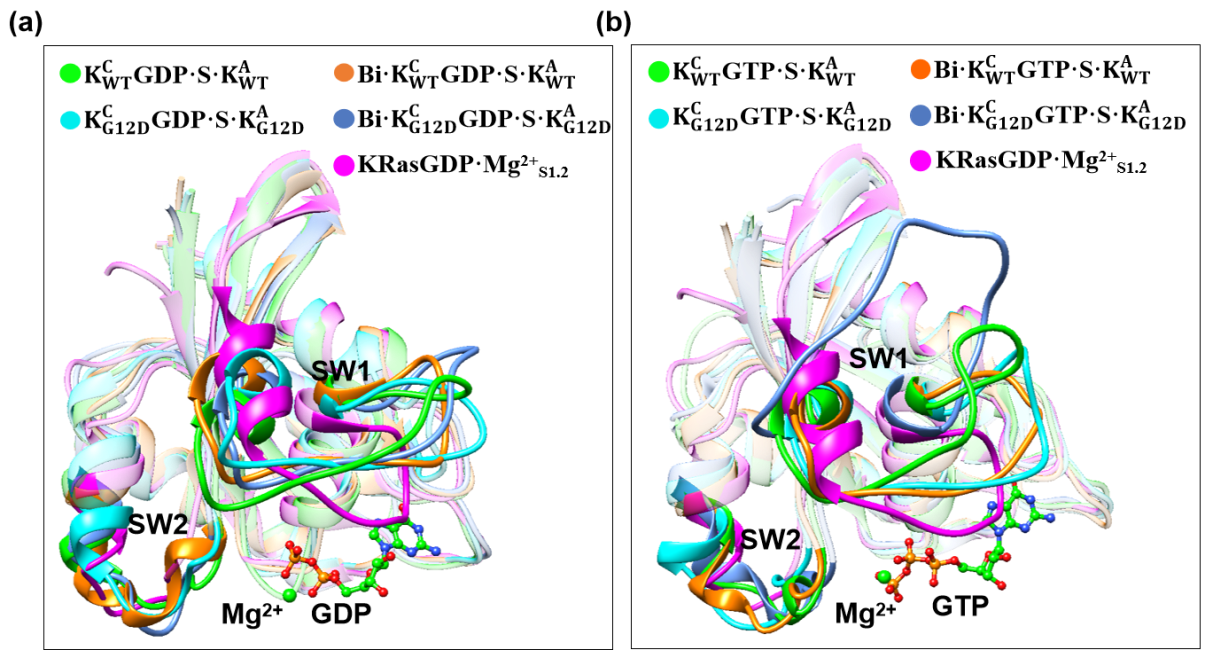


**Figure S5.** The six-coordination of Mg^2+^ ion within KRasGDP (a) and KRasGTP (b) is shown.


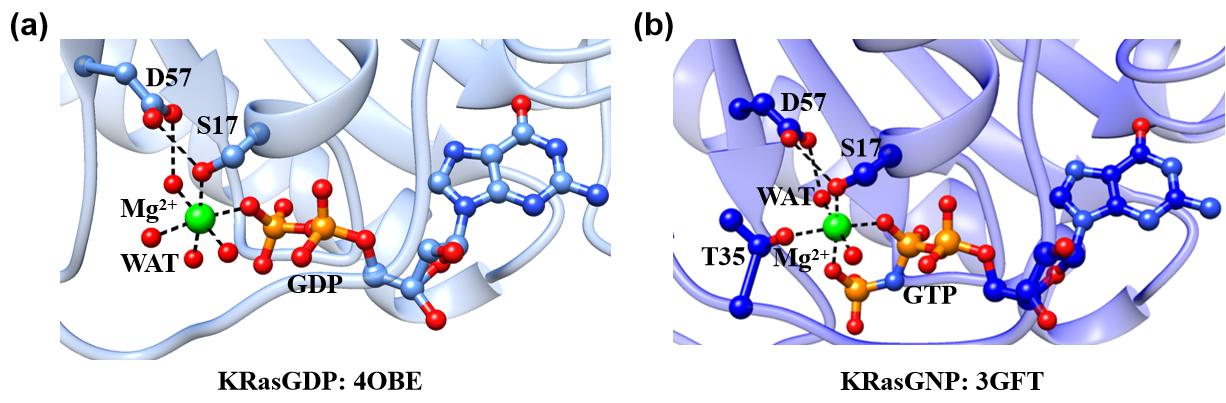


**Figure S6.** (a, c) The heavy-atom RMSD values of GDP from KRas^C^GDP·Mg^2+^ and KRas^C^GTP·Mg^2+^ within ternary complexes function as the simulation time, respectively. Three similar color lines represent RMSD values from three independent simulations. (b, d) The representative structures of KRas^C^GDP·Mg^2+^ and KRas^C^GTP·Mg^2+^ are highlighted with GDP and GTP molecules, respectively.


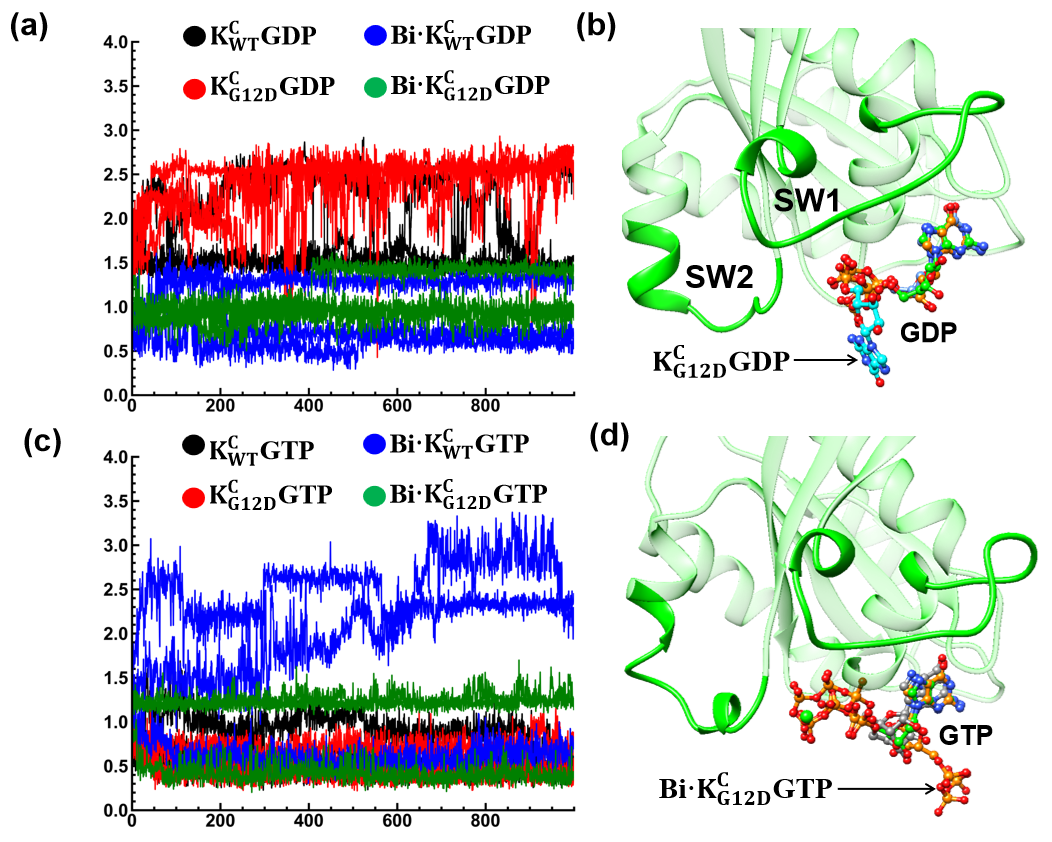


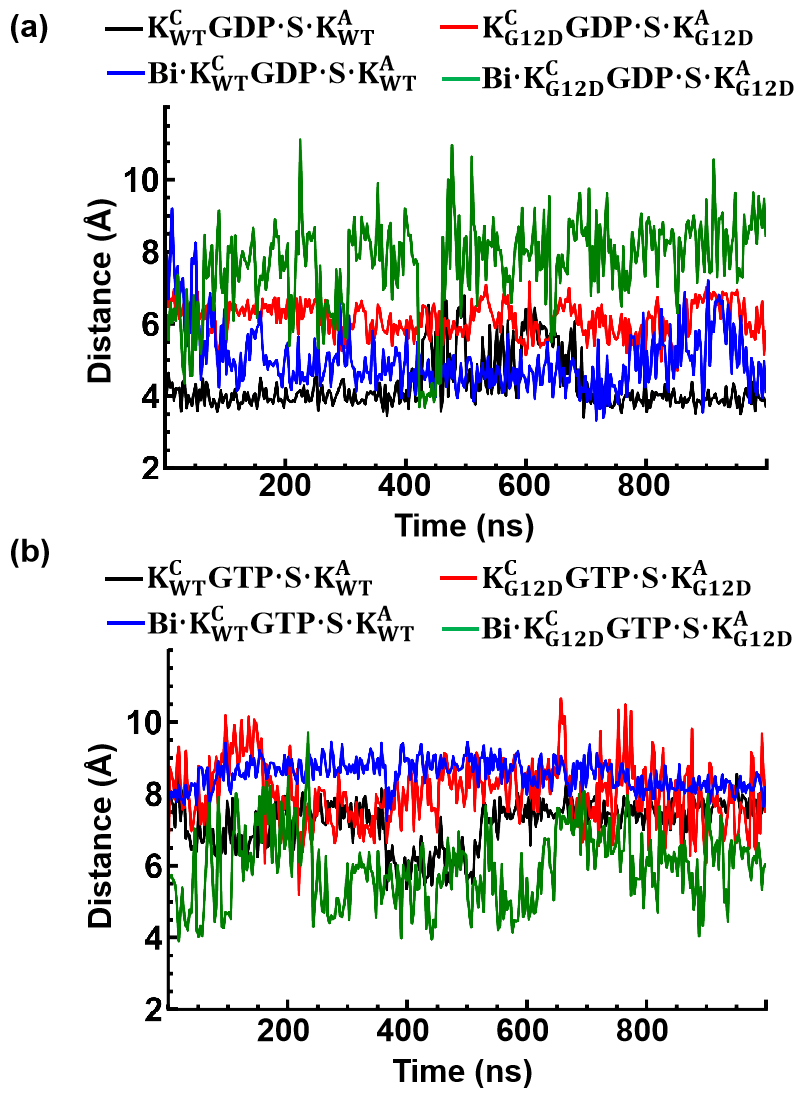
**Figure S7.** The distance between CA atom of D12^K^/G12^K^ and the geometrical center of OE1, OE2 atoms of E62^K^ function as the simulation time in KRas^C^GDP·Mg^2+^ (a) and KRas^C^GTP·Mg^2+^ (b) ternary complexes.

**Figure S8.** (a) The probabilty of three hydrogen bonds (H-bond) are calculated from last 200 ns trajectory. Q70^K^-N879^S^ represents the H-bond between the OE1 atom of Q70 in KRas and the ND2 atom of N879 in SOS1. Bi-N879^S^ is the H-bond between the N3 atom of BI-3406 and the OD1 atom of N879 in SOS1. Bi-M878^S^ shows the H-bond between the N4 atom of BI-3406 and the O atom of M878 in SOS1. (b) The distance between the center of NH1/NH2 atoms in R73 of KRas^C^ and the center of benzene ring in Y884 of SOS1, plotted as a function of simulation time. (c) the distance between the NZ atom of K898 in SOS1 and the center of three F atoms in BI-3406 as a function of simulation time. (d) The distance between the center of CA atoms of F890 and K898 in SOS1 and the center of the quinazoline group in BI-3406 as a function of simulation time.


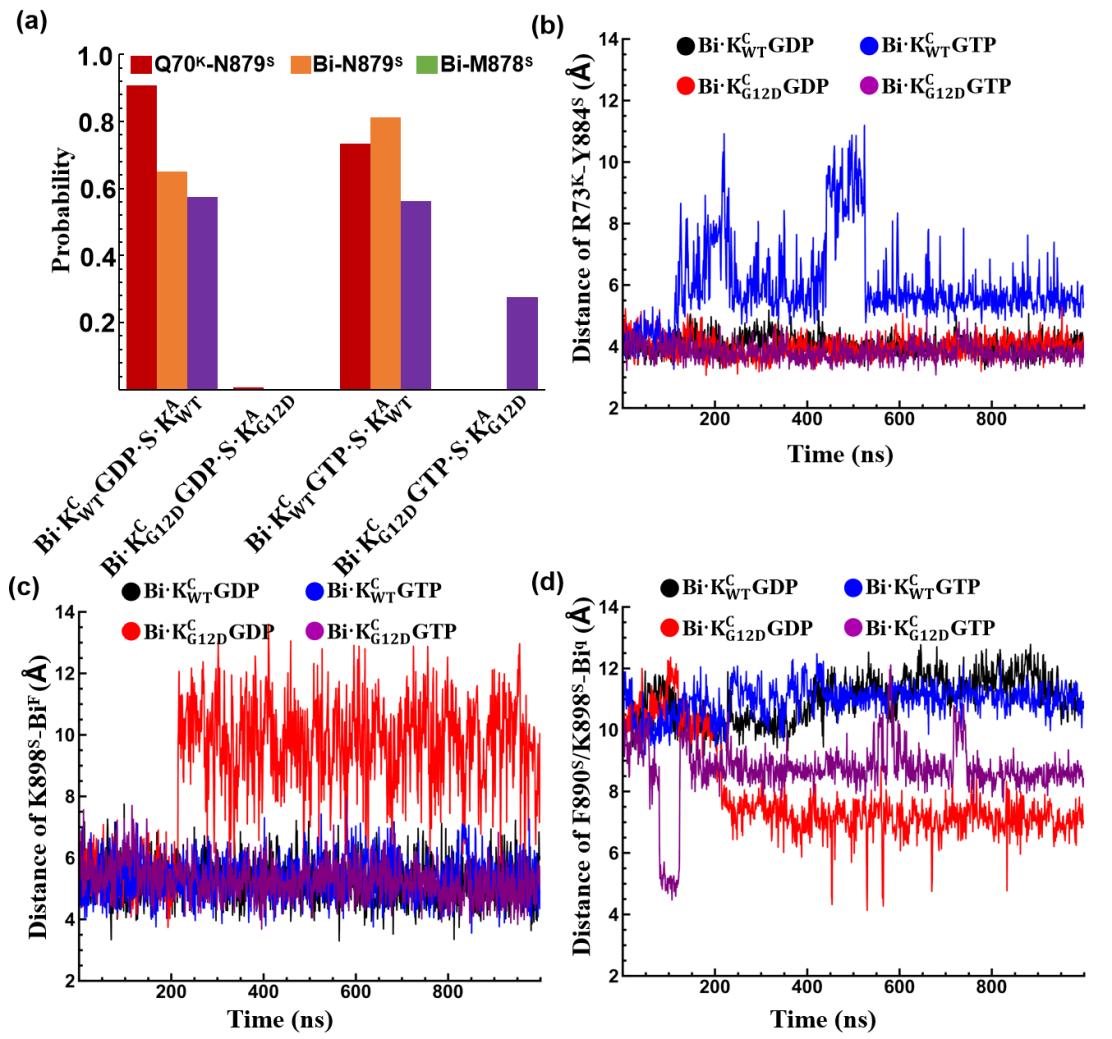


**Figure S9.** The distribution of the dihedral angle N1-C7-N3-C15 in BI-3406.


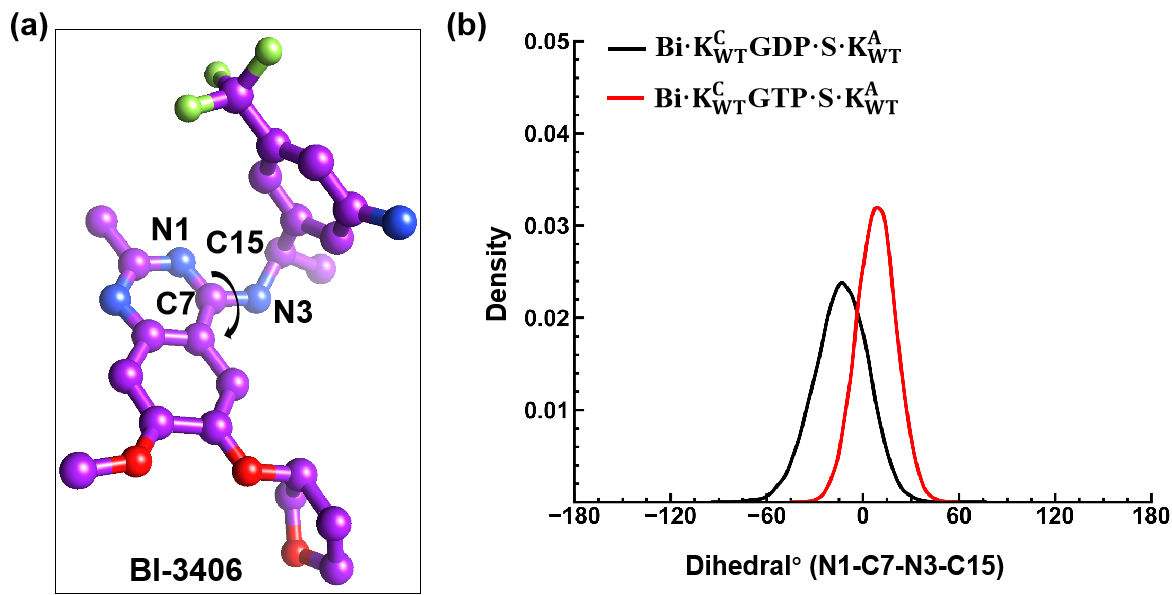


**Figure S10.** The relative binding free energy (∆∆G) in kcal/mo between KRas^C^ and SOSl.


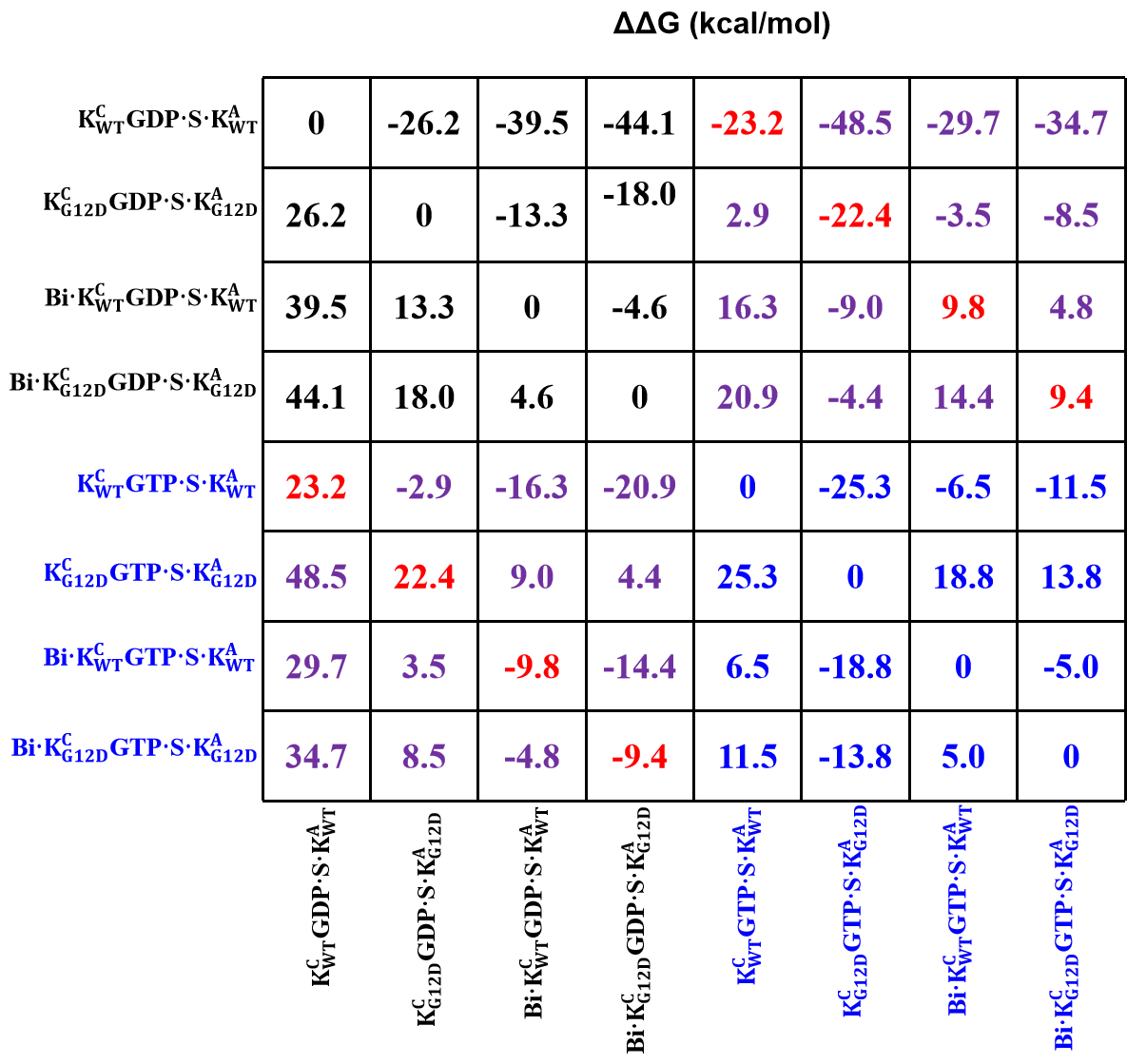


**Figure S11.** The binding affinity with standard deviation between KRasC and SOS1 for each ternary complex is calculated from three independent trajectories with igb=5 and igb=8 terms.


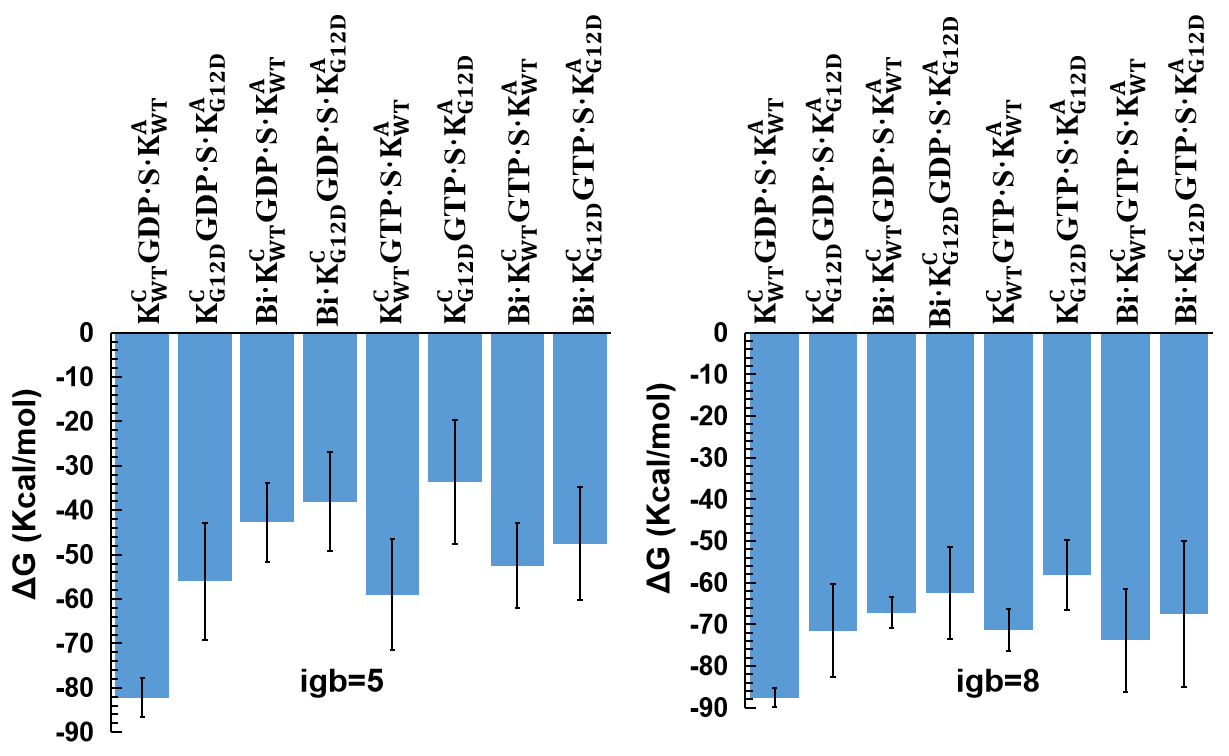

Supplement: S4 Fig — (DOCX) [file pcbi.1014213.s005.docx]
