# Supplementary figures and images for: Computational insights on the molecular interplay between KRas (G12D mutation) and SOS1 modulated by the inhibitor BI-3406
Source: PLoS Comput Biol. 2026 Apr 29;22(4):e1014213. doi: 10.1371/journal.pcbi.1014213 (PMC13155684; doi:10.1371/journal.pcbi.1014213)

**S5 Fig.** The six-coordination of Mg2+ ion within KRasGDP (a) and KRasGTP (b) is shown.


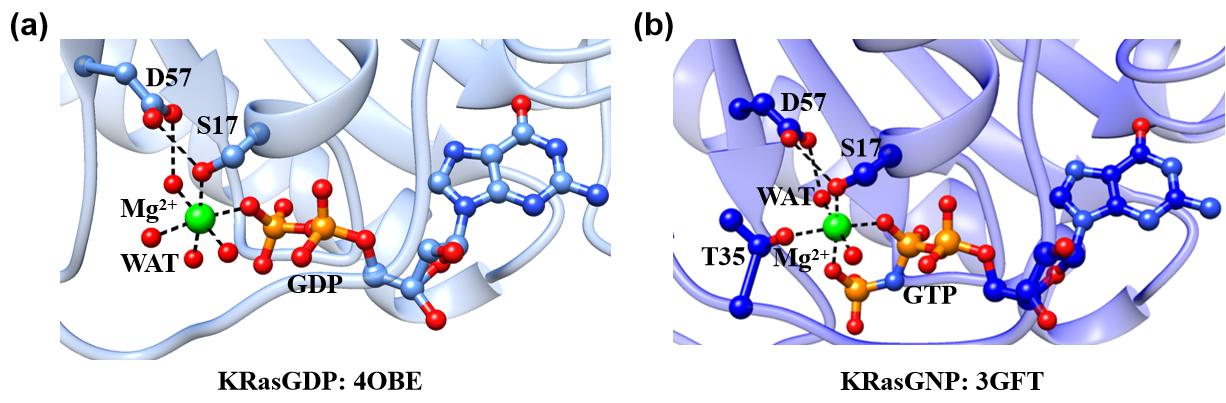

Supplement: S5 Fig — (DOCX) [file pcbi.1014213.s006.docx]

**S9 Fig.** The distribution of the dihedral angle N1-C7-N3-C15 in BI-3406.


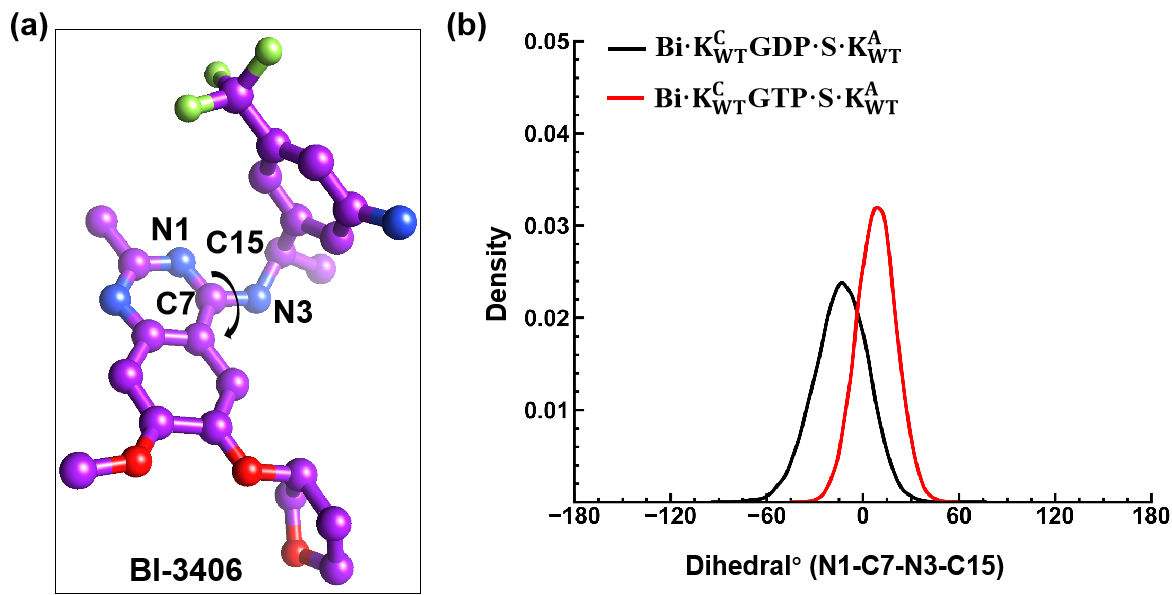

Supplement: S9 Fig — (DOCX) [file pcbi.1014213.s010.docx]

**S10 Fig.** The relative binding free energy (∆∆G) in kcal/mo between KRasC and SOSl.


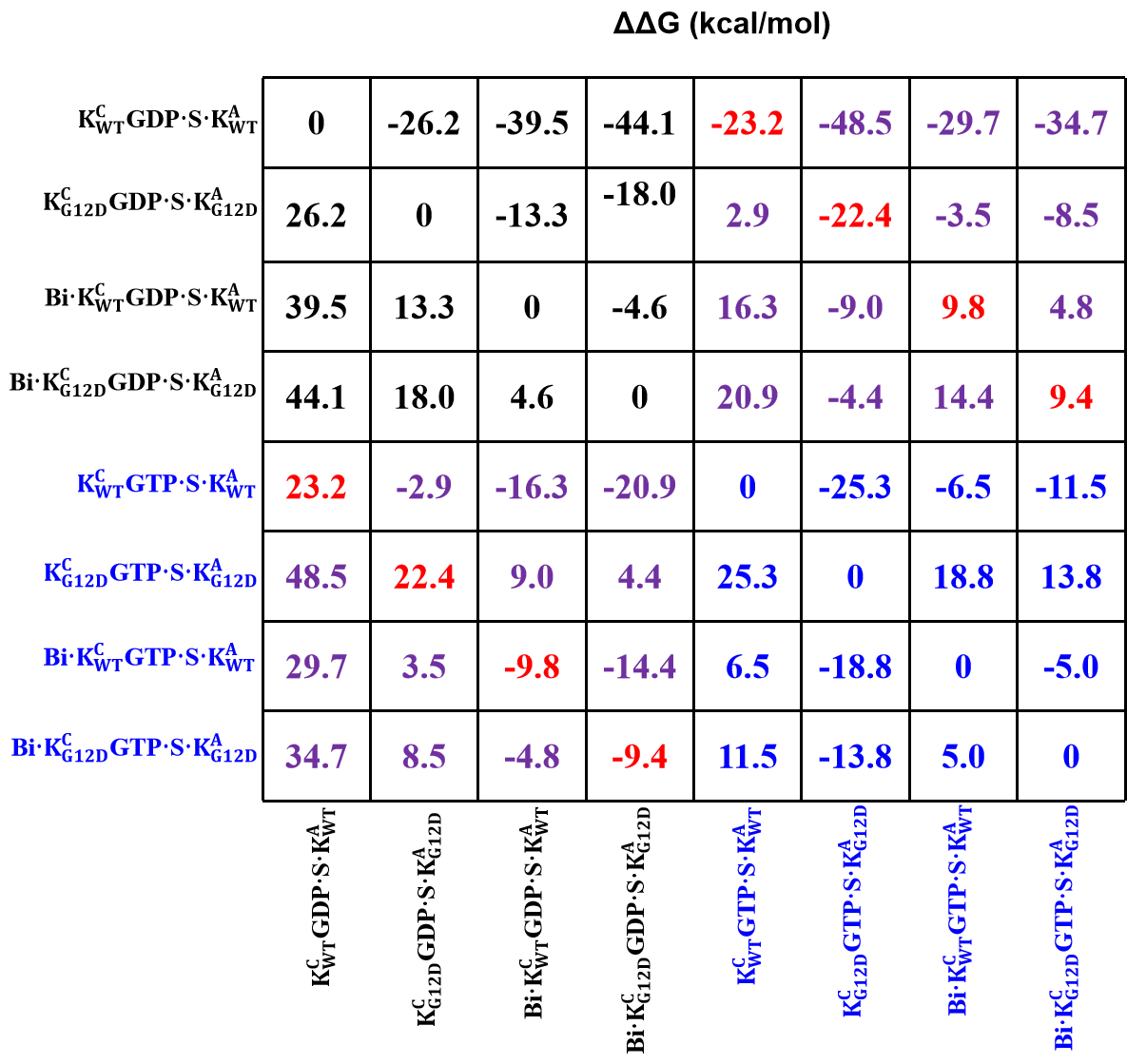

Supplement: S10 Fig — (DOCX) [file pcbi.1014213.s011.docx]
