# Supplementary material for: Computational insights on the molecular interplay between KRas (G12D mutation) and SOS1 modulated by the inhibitor BI-3406
Source: PLoS Comput Biol. 2026 Apr 29;22(4):e1014213. doi: 10.1371/journal.pcbi.1014213 (PMC13155684; doi:10.1371/journal.pcbi.1014213)

**S6 Fig.** (a, c) The heavy-atom RMSD values of GDP from KRas^C^GDP·Mg^2+^ and KRas^C^GTP·Mg^2+^ within ternary complexes function as the simulation time, respectively. Three similar color lines represent RMSD values from three independent simulations. (b, d) The representative structures of KRas^C^GDP·Mg^2+^ and KRas^C^GTP·Mg^2+^ are highlighted with GDP and GTP molecules, respectively.


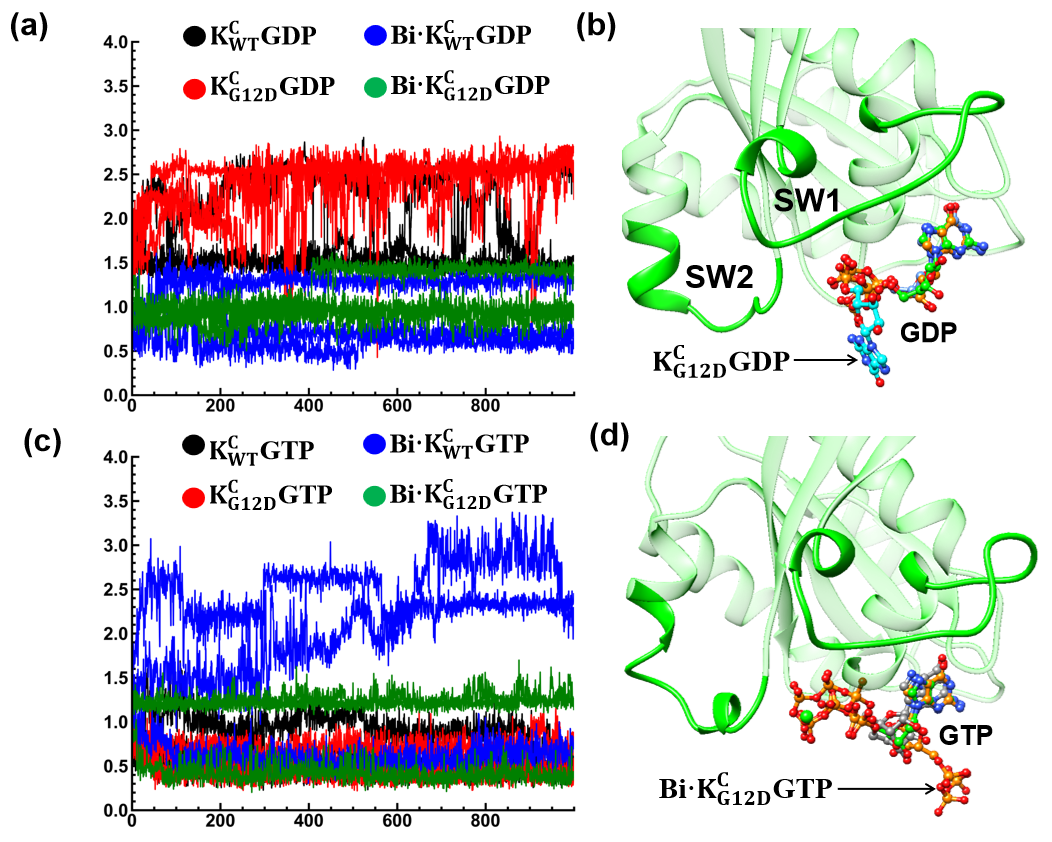

Supplement: S6 Fig — (DOCX) [file pcbi.1014213.s007.docx]
