# Supplementary material for: Computational insights on the molecular interplay between KRas (G12D mutation) and SOS1 modulated by the inhibitor BI-3406
Source: PLoS Comput Biol. 2026 Apr 29;22(4):e1014213. doi: 10.1371/journal.pcbi.1014213 (PMC13155684; doi:10.1371/journal.pcbi.1014213)
Supplement: S7 Fig — (DOCX) [file pcbi.1014213.s008.docx]

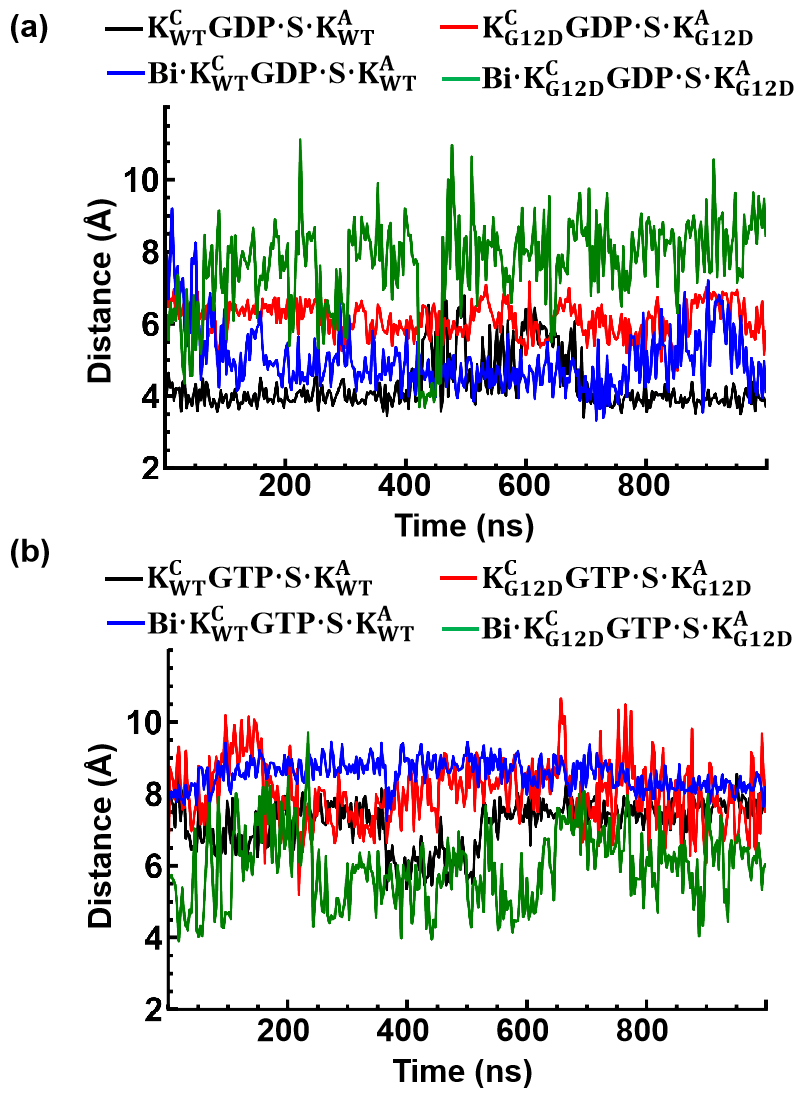
**S7 Fig.** The distance between CA atom of D12^K^/G12^K^ and the geometrical center of OE1, OE2 atoms of E62^K^ function as the simulation time in KRas^C^GDP·Mg^2+^ (a) and KRas^C^GTP·Mg^2+^ (b) ternary complexes.
