# Supplementary material for: Computational insights on the molecular interplay between KRas (G12D mutation) and SOS1 modulated by the inhibitor BI-3406
Source: PLoS Comput Biol. 2026 Apr 29;22(4):e1014213. doi: 10.1371/journal.pcbi.1014213 (PMC13155684; doi:10.1371/journal.pcbi.1014213)

**S8 Fig.** (a) The probabilty of three hydrogen bonds (H-bond) are calculated from last 200 ns trajectory. Q70^K^-N879^S^ represents the H-bond between the OE1 atom of Q70 in KRas and the ND2 atom of N879 in SOS1. Bi-N879^S^ is the H-bond between the N3 atom of BI-3406 and the OD1 atom of N879 in SOS1. Bi-M878^S^ shows the H-bond between the N4 atom of BI-3406 and the O atom of M878 in SOS1. (b) The distance between the center of NH1/NH2 atoms in R73 of KRas^C^ and the center of benzene ring in Y884 of SOS1, plotted as a function of simulation time. (c) the distance between the NZ atom of K898 in SOS1 and the center of three F atoms in BI-3406 as a function of simulation time. (d) The distance between the center of CA atoms of F890 and K898 in SOS1 and the center of the quinazoline group in BI-3406 as a function of simulation time.


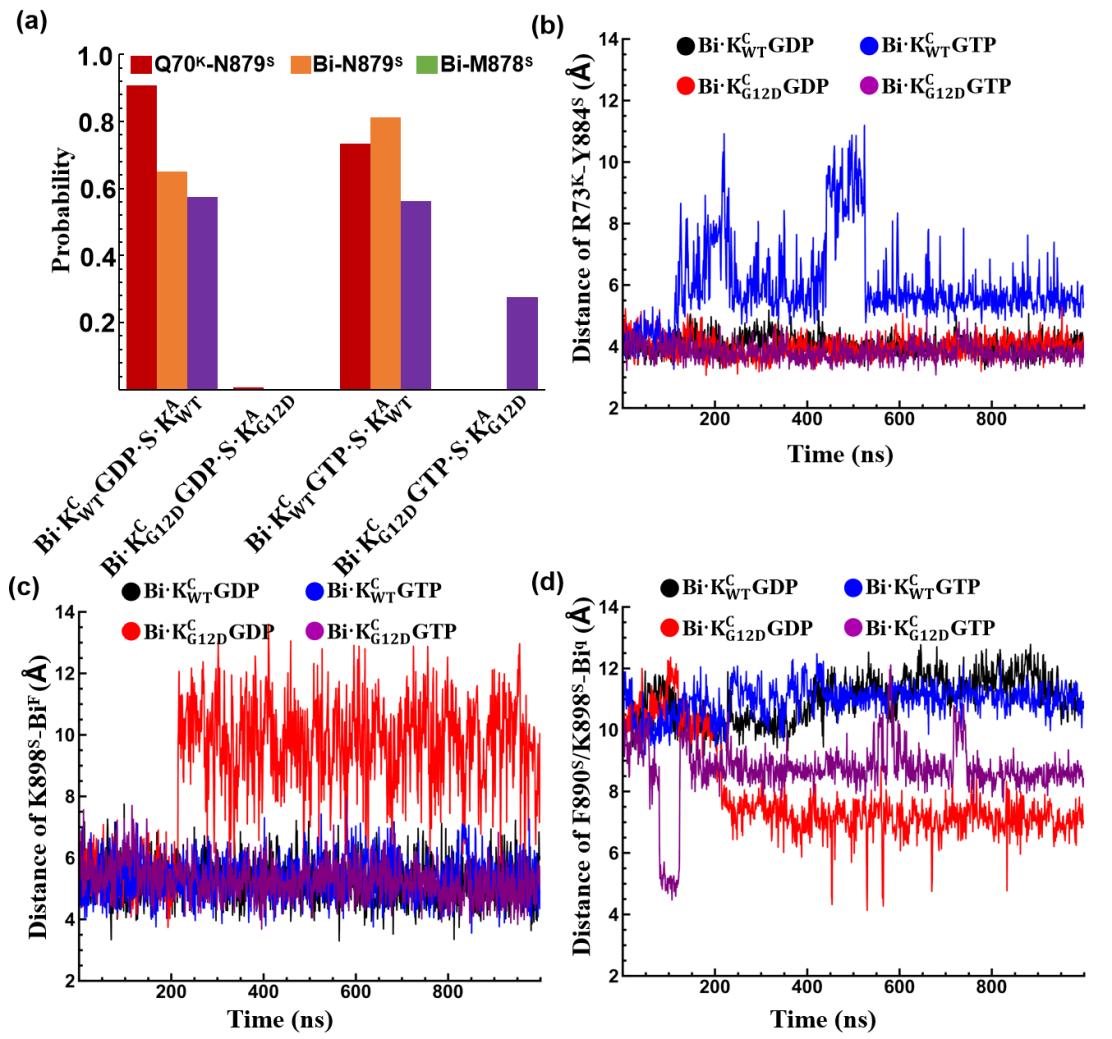

Supplement: S8 Fig — (DOCX) [file pcbi.1014213.s009.docx]
