# Supplementary material for: Computational insights on the molecular interplay between KRas (G12D mutation) and SOS1 modulated by the inhibitor BI-3406
Source: PLoS Comput Biol. 2026 Apr 29;22(4):e1014213. doi: 10.1371/journal.pcbi.1014213 (PMC13155684; doi:10.1371/journal.pcbi.1014213)

**S11 Fig.** The binding affinity with standard deviation between KRasC and SOS1 for each ternary complex is calculated from three independent trajectories with igb=5 and igb=8 terms.


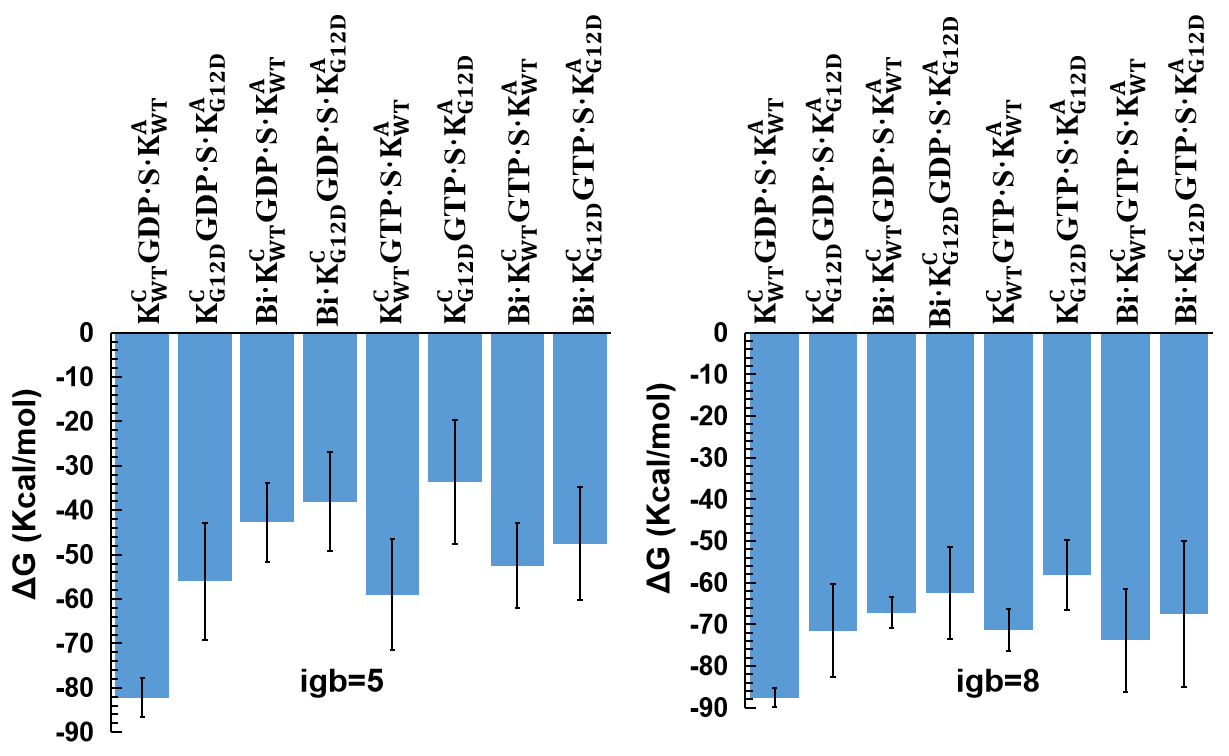

Supplement: S11 Fig — (DOCX) [file pcbi.1014213.s012.docx]
